# Supplementary material for: A unifying framework for mean-field theories of asymmetric kinetic Ising systems
Source: Nat Commun. 2021 Feb 19;12:1197. doi: 10.1038/s41467-021-20890-5 (PMC7895831; doi:10.1038/s41467-021-20890-5)
Supplement: Supplementary file 1 — Supplementary Information [file 41467_2021_20890_MOESM1_ESM.pdf]

A unifying framework for mean-field theories of asymmetric kinetic Ising systems: Supplementary Information

Miguel Aguilera,* S. Amin Moosavi, and Hideaki Shimazaki

Supplementary Note 1: General approach for Plefka expansions

Let $\mathbf{s}_t = \{s_{i,t}\}_i,\ i = 1, \ldots, N$ be the state of the system at time t and $\mathbf{s}_{1:T} = \{\mathbf{s}_{i,t}\}_{i,t},\ i = 1, \ldots, N,\ t = 1, \ldots, T$ a trajectory of the system. Given an initial state $\mathbf{s}_0$, the probability of a trajectory $\mathbf{s}_{1:T}$ of a kinetic Ising model is:

$$P(\mathbf{s}_{1:T}) = \prod_t P(\mathbf{s}_t|\mathbf{s}_{t-1}) = \prod_t \exp\left(\sum_i s_{i,t} h_{i,t} - \psi\right), \tag{1}$$

$$h_{i,t} = H_i + \sum_j J_{ij} s_{j,t-1}, \tag{2}$$

$$\psi = \sum_{t,i} \log 2\cosh h_{i,t}. \tag{3}$$

Note that ψ depends on the specific trajectory $\mathbf{s}_{0:T}$. The manifold $\mathcal{P} = \{P(\mathbf{s}_{1:T}|\mathbf{H}, \mathbf{J})\}$ defines the family of probability distributions of the trajectories of all kinetic Ising models. Within this manifold, we consider a submanifold $\mathcal{P}_0$ in which the probability distributions of a set of elements of the system $\{\{s_{i,t}\}_{i\in\mathcal{I}_t}\}_t$ is tractable and independent from the rest of the elements of the system. Here $\mathcal{I}_t$ denotes the indices of the tractable elements of the system at time t. Trajectories in the submanifold are defined as:

$$P_0(\mathbf{s}_{1:T}) = \prod_t P_0(\mathbf{s}_t|\mathbf{s}_{1:t-1}) = \prod_t \exp\left(\sum_{i\in\mathcal{I}_t} s_{i,t}\theta_{i,t} + \sum_{i\in\overline{\mathcal{I}}_t} s_{i,t} h_{i,t} - \psi_0\right), \tag{4}$$

$$\psi_0 = \sum_{t,i\in\mathcal{I}_t} \log 2\cosh \theta_{i,t} + \sum_{t,i\in\overline{\mathcal{I}}_t} \log 2\cosh h_{i,t}, \tag{5}$$

where $\overline{\mathcal{I}}_t$ is the complement set of $\mathcal{I}_t$ for the elements at time t. The mean-field definition of the new effective field is $\theta_{i,t} = \Theta_{i,t}$, although other definitions are possible. We restrict the function $\theta_{i,t}$ to maximum caliber models composed of individual fields and pairwise couplings

$$\theta_{i,t} = \sum_{I\in\mathcal{C}_{i,t}} \Theta_{i,t,I} s_I, \tag{6}$$

where $\mathcal{C}_{i,t}$ is a set of couplings $\{(j,\tau)\}, \tau < t, j \in \mathcal{I}_\tau$ between $s_{i,t}$ and other nodes $s_{j,\tau}$ from the set of nodes with tractable properties. We define the individual field of a unit with $\Theta_{i,t,\emptyset} \equiv \Theta_{i,t}$, by defining $s_\emptyset = 1$. In general, the effect from the past spiking activities at $\tau < t$ can be modeled by this equation, which includes the generalized linear model for conditionally independent Bernoulli processes. In the approximations explored in this paper, however, we focus on the effect from the immediate past $\tau = t - 1$.

Different approximations are defined through different definitions of $\theta_{i,t}$ using a model connecting P and P_0. This model is defined through a parameter α:

$$P_\alpha(\mathbf{s}_{1:T}) = \prod_t P_\alpha(\mathbf{s}_t|\mathbf{s}_{1:t-\tau-1}) = \prod_t \exp\left(\sum_{i\in\mathcal{I}_t} s_{i,t}((1-\alpha)\theta_{i,t} + \alpha h_{i,t}) + \sum_{i\in\overline{\mathcal{I}}_t} s_{i,t} h_{i,t} - \psi_\alpha\right), \tag{7}$$

$$\psi_\alpha = \sum_{t,i\in\mathcal{I}_t} \log 2\cosh((1-\alpha)\theta_{i,t} + \alpha h_{i,t})) + \sum_{t,i\in\overline{\mathcal{I}}_t} \log 2\cosh h_{i,t}, \tag{8}$$

* sci@maguilera.net

such that $P_1(\mathbf{s}_{1:T}) = P(\mathbf{s}_{1:T})$.

The model P_0 that better approximates P is the one that minimizes the Kullback Leibler divergence:

$$D_{KL}(P||P_0) = \sum_{\mathbf{s}_{1:T}} P(\mathbf{s}_{1:T}) \log \frac{P(\mathbf{s}_{1:T})}{P_0(\mathbf{s}_{1:T})}. \tag{9}$$

Thus its parameters $\Theta_{i,t,I}$ meet

$$\frac{\partial D_{KL}(P||P_0)}{\partial \Theta_{i,t,I}} = -\sum_{\mathbf{s}_{1:T}} P(\mathbf{s}_{1:T}) s_{i,t} s_I + \sum_{\mathbf{s}_{1:T}} P(\mathbf{s}_{1:T}) \sum_{\boldsymbol{\sigma}_t} P_0(\boldsymbol{\sigma}_t|\mathbf{s}_{1:t-1}) \sigma_{i,t} s_I \tag{10}$$

$$= \langle s_{i,t} s_I \rangle_0^t - \langle s_{i,t} s_I \rangle_1^t = 0, \tag{11}$$

where $\langle \dots \rangle_\alpha^t = \sum_{\mathbf{s}_{1:T}} \dots P_\alpha(\mathbf{s}_t|\mathbf{s}_{1:t-1}) P(\mathbf{s}_{1:t-1})$. Thus, the closest approximation P_0 to P is the one in which $\langle s_{i,t} s_I \rangle_0^t = \langle s_{i,t} s_I \rangle_1^t, \forall i, I$. Furthermore, models P_0 are tractable for indices $\{\mathcal{I}_t\}$, in the sense that knowing $\Theta_{i,t,I}$, it is easy to compute $\langle s_{i,t} s_I \rangle_0^t$. The value of parameters $\Theta_{i,t,I}$ cannot be computed directly in general, but they can be approximated by computing a Plefka expansion. Approximating $\langle s_{i,t} s_I \rangle_1^t$ by the Taylor expansion of $\langle s_{i,t} s_I \rangle_\alpha^t$ from $\alpha = 0$, we have

$$\langle s_{i,t} s_I \rangle_\alpha^t = \langle s_{i,t} s_I \rangle_{\alpha=0}^t + \sum_{k=1}^n \frac{\alpha^k}{k!} \frac{\partial^k \langle s_{i,t} s_I \rangle_{\alpha=0}^t}{\partial \alpha^k} + \mathcal{O}(\alpha^{(n+1)}). \tag{12}$$

Evaluating it at $\alpha = 1$ knowing that $\langle s_{i,t} s_I \rangle_0^t = \langle s_{i,t} s_I \rangle_1^t, \forall i, I$, we have

$$\sum_{k=1}^n \left[\frac{\alpha^k}{k!} \right]_{\alpha=1} \frac{\partial^k \langle s_{i,t} s_I \rangle_0^t}{\partial \alpha^k} = 0 + \left[\mathcal{O}(\alpha^{(n+1)}) \right]_{\alpha=1}. \tag{13}$$

Let us define $\Delta h_{i,t} = -\theta_{i,t} + h_{i,t}$, we have that

$$\frac{\partial P_\alpha(\mathbf{s})}{\partial \alpha} = \sum_t \sum_{i \in \mathcal{I}_t} \left(s_{i,t} \Delta h_{i,t} - \langle s_{i,t} \Delta h_{i,t} \rangle_{t,\alpha} \right) P_\alpha(\mathbf{s}), \tag{14}$$

$$\frac{\partial^2 P_\alpha(\mathbf{s})}{\partial \alpha^2} = \left(\sum_t \sum_{i \in \mathcal{I}_t} s_{i,t} \Delta h_{i,t} - \langle s_{i,t} \Delta h_{i,t} \rangle_{t,\alpha} \right)^2 P_\alpha(\mathbf{s}) \tag{15}$$

$$- \sum_t \left(\left\langle \left(\sum_{i \in \mathcal{I}_t} s_{i,t} \Delta h_{i,t} \right)^2 \right\rangle_{t,\alpha} - \left\langle \sum_{i \in \mathcal{I}_t} s_{i,t} \Delta h_{i,t} \right\rangle_{t,\alpha}^2 \right) P_\alpha(\mathbf{s}) \tag{16}$$

$$= \sum_t \left(\left(\left(\sum_{i \in \mathcal{I}_t} s_{i,t} \Delta h_{i,t} \right)^2 - \left\langle \left(\sum_{i \in \mathcal{I}_t} s_{i,t} \Delta h_{i,t} \right)^2 \right\rangle_{t,\alpha} \right) \right. \tag{17}$$

$$\left. - 2 \left\langle \sum_{i \in \mathcal{I}_t} s_{i,t} \Delta h_{i,t} \right\rangle_{t,\alpha} \left(\sum_{k \in \mathcal{I}_t} s_{k,t} \Delta h_{k,t} - \left\langle \sum_{k \in \mathcal{I}_t} s_{k,t} \Delta h_{k,t} \right\rangle_{t,\alpha} \right) \right) P_\alpha(\mathbf{s}), \tag{18}$$

where $\langle \dots \rangle_{t,\alpha} = \sum_{\mathbf{s}_t} \dots P_\alpha(\mathbf{s}_t|\mathbf{s}_{1:t-1})$. From these equations we derive the first and second order approximations.

For the first order term, we have

$$\frac{\partial \langle s_{i,t} s_I \rangle_{\alpha=0}^t}{\partial \alpha} = \sum_{\mathbf{s}_{1:T}} s_{i,t} s_I \frac{\partial P_0(\mathbf{s})}{\partial \alpha} \tag{19}$$

$$= \sum_{\mathbf{s}_{1:T}} \left(\sum_t \sum_{k \in \mathcal{I}_t} \left(s_{i,t} s_I (s_{k,t} \Delta h_{k,t} - \langle s_{k,t} \Delta h_{k,t} \rangle_{t,0}) \right) \right) P_0(\mathbf{s}). \tag{20}$$

The second order term is:

$$\frac{\partial^2 \langle s_{i,t} s_I \rangle^t_{\alpha=0}}{\partial \alpha^2} = \sum_{\mathbf{s}_{1:T}} s_{i,t} s_I \frac{\partial^2 P_0(\mathbf{s})}{\partial \alpha^2} \tag{21}$$

$$= \sum_{\mathbf{s}_{1:T}} s_{i,t} s_I \left(\left(\sum_{k \in \mathcal{I}_t} s_{k,t} \Delta h_{k,t} \right)^2 - \left\langle \left(\sum_{k \in \mathcal{I}_t} s_{k,t} \Delta h_{k,t} \right)^2 \right\rangle_{t,0} \right. \tag{22}$$

$$\left. - 2 \left\langle \sum_{k \in \mathcal{I}_t} s_{k,t} \Delta h_{k,t} \right\rangle_{t,0} \left(\sum_{m \in \mathcal{I}_t} s_{m,t} \Delta h_{m,t} - \left\langle \sum_{m \in \mathcal{I}_t} s_{m,t} \Delta h_{m,t} \right\rangle_{t,0} \right) \right) P_0(\mathbf{s}). \tag{23}$$

Using the equations above to solve Supplementary Eq. 13 for different orders and choices of P_0 will give us the different Plekfa approximations.

Supplementary Note 2: Plefka$[t-1,t]$

This approximation uses the following approximated marginal probability distribution:

$$P_\alpha^{[t-1:t]}(\mathbf{s}_t,\mathbf{s}_{t-1}) = \sum_{\mathbf{s}_{t-2}} P_\alpha(\mathbf{s}_t|\mathbf{s}_{t-1}) P_\alpha(\mathbf{s}_{t-1}|\mathbf{s}_{t-2}) P(\mathbf{s}_{t-2}), \tag{24}$$

where

$$P_\alpha(s_{i,t}|\mathbf{s}_{t-1}) = \frac{\mathrm{e}^{s_{i,t}h_{i,t}(\alpha)}}{2\cosh h_{i,t}(\alpha)}, \tag{25}$$

$$h_{i,t}(\alpha) = (1-\alpha)\Theta_{i,t} + \alpha(H_i + \sum_j J_{ij}s_{j,t-1}). \tag{26}$$

Here, by increasing the value of α from 0 to 1, one can smoothly connect the independent and coupled models. Further, $h_{i,t}(\alpha)$ can be written as

$$h_{i,t}(\alpha) = \Theta_{i,t} + \alpha\,\Delta h_{i,t}, \tag{27}$$

where $\Delta h_{i,t} = -\Theta_{i,t} + H_i + \sum_j J_{ij}s_{j,t-1}$ represents deviation from the independent model. We estimate $m_{i,t}$ by using its α-dependent approximation, defined as

$$m_{i,t}(\alpha) = \sum_{\mathbf{s}_t,\mathbf{s}_{t-1}} s_{i,t} P_\alpha^{[t-1:t]}(\mathbf{s}_t,\mathbf{s}_{t-1}) = \sum_{\mathbf{s}_{t-1},\mathbf{s}_{t-2}} \tanh h_{i,t}(\alpha) P_\alpha(\mathbf{s}_{t-1}|\mathbf{s}_{t-2}) P(\mathbf{s}_{t-2}). \tag{28}$$

Approximating its value by expanding around $\alpha = 0$ yields

$$m_{i,t}(\alpha) = m_{i,t}(\alpha=0) + \sum_{k=1}^{n} \frac{\alpha^k}{k!}\frac{\partial^k m_{i,t}(\alpha=0)}{\partial\alpha^k} + \mathcal{O}(\alpha^{(n+1)}), \tag{29}$$

By noting that $m_{i,t}(\alpha=0) = m_{i,t}(\alpha=1)$, we evaluate it at $\alpha = 1$. This results in

$$\left[\sum_{k=1}^{n} \frac{\alpha^k}{k!}\frac{\partial^k m_{i,t}(\alpha=0)}{\partial\alpha^k}\right]_{\alpha=1} = 0 + \left[\mathcal{O}(\alpha^{(n+1)})\right]_{\alpha=1}. \tag{30}$$

The approximation yields the nMF equations when we ignore quadratic and higher order terms in solving this equation, and the TAP equations when ignoring third and higher order terms.

The first order derivative of $m_{i,t}(\alpha)$ is given as

$$\frac{\partial m_{i,t}(\alpha)}{\partial\alpha} = \sum_{\mathbf{s}_{t-1},\mathbf{s}_{t-2}} \left[\frac{\partial \tanh h_{i,t}(\alpha)}{\partial\alpha} P_\alpha(\mathbf{s}_{t-1}|\mathbf{s}_{t-2}) + \tanh h_{i,t}(\alpha)\frac{\partial P_\alpha(\mathbf{s}_{t-1}|\mathbf{s}_{t-2})}{\partial\alpha}\right] P(\mathbf{s}_{t-2}). \tag{31}$$

Using the following equation,

$$\frac{\partial \tanh h_{i,t}(\alpha)}{\partial\alpha} = (1-\tanh^2 h_{i,t}(\alpha))\Delta h_{i,t}, \tag{32}$$

the first order derivative is given as

$$\frac{\partial m_{i,t}(\alpha)}{\partial\alpha} = \sum_{\mathbf{s}_{t-1},\mathbf{s}_{t-2}} \left[(1-\tanh^2 h_{i,t}(\alpha))\Delta h_{i,t} + \tanh h_{i,t}(\alpha)\frac{\partial P_\alpha(\mathbf{s}_{t-1}|\mathbf{s}_{t-2})}{\partial\alpha}\right] P_\alpha(\mathbf{s}_{t-1}|\mathbf{s}_{t-2}) P(\mathbf{s}_{t-2}). \tag{33}$$

Expectation of the first term at $\alpha = 0$ is

$$\sum_{\mathbf{s}_{t-1},\mathbf{s}_{t-2}} (1-\tanh^2 h_{i,t}(0))\Delta h_{i,t} P_0(\mathbf{s}_{t-1}) = (1-m_{i,t}^2)(-\Theta_{i,t} + H_i + \sum_j J_{ij}m_{j,t-1}). \tag{34}$$

The second term becomes zero at $\alpha = 0$, since the derivative of $P_\alpha(\mathbf{s}_{t-1}|\mathbf{s}_{t-2})$ becomes independent of $\tanh h_{i,t}(\alpha)$ for $\alpha = 0$ and we know that $\sum_{\mathbf{s}_{t-1}} P_\alpha(\mathbf{s}_{t-1}|\mathbf{s}_{t-2}) = 1$. Thus we have

$$\frac{\partial m_{i,t}(\alpha=0)}{\partial \alpha} = (1-m_{i,t}^2)(-\Theta_{i,t} + H_i + \sum_j J_{ij} m_{j,t-1}). \tag{35}$$

From here, we obtain the nMF equations, yielding $\Big[\alpha(-\Theta_{i,t} + H_i + \sum_j J_{ij} m_{j,t-1})\Big]_{\alpha=1} = 0 + \big[\mathcal{O}(\alpha^2)\big]_{\alpha=1}$ and

$$\Theta_{i,t} = H_i + \sum_j J_{ij} m_{j,t-1} + \big[\mathcal{O}(\alpha^1)\big]_{\alpha=1}, \tag{36}$$

$$m_{i,t} \approx \tanh[H_i + \sum_j J_{ij} m_{j,t-1}]. \tag{37}$$

The second order derivative of $m_{i,t}(\alpha)$ is given as

$$\frac{\partial^2 m_{i,t}(\alpha)}{\partial \alpha^2} = \sum_{\mathbf{s}_{t-1},\mathbf{s}_{t-2}} \Bigg[\frac{\partial^2 \tanh h_{i,t}(\alpha)}{\partial \alpha^2} P_\alpha(\mathbf{s}_{t-1}|\mathbf{s}_{t-2}) + 2 \frac{\partial \tanh h_{i,t}(\alpha)}{\partial \alpha} \frac{\partial P_\alpha(\mathbf{s}_{t-1}|\mathbf{s}_{t-2})}{\partial \alpha} \tag{38}$$

$$+ \tanh h_{i,t}(\alpha) \frac{\partial^2 P_\alpha(\mathbf{s}_{t-1}|\mathbf{s}_{t-2})}{\partial \alpha^2} \Bigg] P(\mathbf{s}_{t-2}). \tag{39}$$

Here we note that

$$\frac{\partial^2 \tanh h_{i,t}(\alpha)}{\partial \alpha^2} = -2 \tanh h_{i,t}(\alpha)(1-\tanh^2 h_{i,t}(\alpha)) \Delta h_{i,t}^2. \tag{40}$$

From these equations, the second derivative is computed as

$$\frac{\partial^2 m_{i,t}(\alpha)}{\partial \alpha^2} = \sum_{\mathbf{s}_{t-1},\mathbf{s}_{t-2}} \Big(-2 \tanh h_{i,t}(\alpha)(1-\tanh^2 h_{i,t}(\alpha)) \Delta h_{i,t}^2 P_\alpha(\mathbf{s}_{t-1}|\mathbf{s}_{t-2}) \tag{41}$$

$$+ 2(1-\tanh^2 h_{i,t}(\alpha)) \Delta h_{i,t} \frac{\partial P_\alpha(\mathbf{s}_{t-1}|\mathbf{s}_{t-2})}{\partial \alpha} \tag{42}$$

$$+ \frac{\partial^2 P_\alpha(\mathbf{s}_{t-1}|\mathbf{s}_{t-2})}{\partial \alpha^2} \Big) P(\mathbf{s}_{t-2}). \tag{43}$$

We evaluate the second derivative at $\alpha = 0$. Here at $\alpha = 0$ the third term is zero since $\sum_{\mathbf{s}_{t-1}} P_\alpha(\mathbf{s}_{t-1}|\mathbf{s}_{t-2}) = 1$, so its derivatives are equal to zero. Thus we have

$$\frac{\partial^2 m_{i,t}(\alpha=0)}{\partial \alpha^2} = -2 m_{i,t}(1-m_{i,t}^2)((-\Theta_{i,t} + H_i + \sum_j J_{ij} m_{j,t-1})^2 + \sum_j J_{ij}^2 (1-m_{j,t-1}^2)) \tag{44}$$

$$+ 2(1-m_{i,t}^2) \sum_j J_{ij} \frac{\partial m_{j,t-1}(\alpha)}{\partial \alpha}, \tag{45}$$

where the last term comes from the $s_{j,t-1}$ terms in $h_{i,t}(\alpha)$ multiplied by $\frac{\partial P_\alpha(\mathbf{s}_{t-1}|\mathbf{s}_{t-2})}{\partial \alpha}$.

Note that the second order term in Supplementary Eq. 45 contains the expression $\Big[\alpha^2(-\Theta_{i,t} + H_i + \sum_j J_{ij} m_{j,t-1})^2\Big]_{\alpha=1}$ and $\Big[\alpha^2 \frac{\partial m_{k,t-1}(\alpha)}{\partial \alpha}\Big]_{\alpha=1}$ that can be neglected as terms with order higher than quadratic. This is due to the fact that the second-order approximation is in the proximity of naive mean-field solution which is in the first order of α. Therefore, we know that $\Big[\alpha(1-m_i^2)(-\Theta_{i,t} + H_i + \sum_j J_{ij} m_{j,t-1})\Big]_{\alpha=1} = \big[\mathcal{O}(\alpha^2)\big]_{\alpha=1}$ and thus $\Big[\alpha^2(-\Theta_{i,t} + H_i + \sum_j J_{ij} m_{j,t-1})^2\Big]_{\alpha=1} = \big[\mathcal{O}(\alpha^4)\big]_{\alpha=1}$ and $\Big[\alpha^2 \frac{\partial m_{k,t-1}(\alpha)}{\partial \alpha}\Big]_{\alpha=1} = \big[\mathcal{O}(\alpha^3)\big]_{\alpha=1}$ which can be neglected for the second order approximation.

The combination of the first and second order derivatives of $m_{i,t}$ evaluated at $\alpha = 0$ allows to solve Supplementary Eq. 30 for order $n = 2$, yielding the TAP equations:

$$\Theta_{i,t} = H_i + \sum_j J_{ij} m_{j,t-1} - m_{i,t} \sum_{jl} J_{ij}^2 (1-m_{j,t-1}^2) + \big[\mathcal{O}(\alpha^2)\big]_{\alpha=1}, \tag{46}$$

$$m_{i,t} \approx \tanh[H_i + \sum_j J_{ij} m_{j,t-1} - m_{i,t} \sum_{jl} J_{ij}^2 (1-m_{j,t-1}^2)]. \tag{47}$$

These results have a form similar to the TAP equations obtained for symmetric and asymmetric networks [1, 2].

Equal-time correlations

Here we approximate $C_{ik,t}$ by evaluating the Plefka expansion of $C_{ik,t}(\alpha = 1)$ around $\alpha = 0$.

When $i = k$, we have $C_{ii,t}(\alpha) = 1 - m_{i,t}(\alpha)^2$. When $i \neq k$, correlations in the system can be obtained by expanding the alpha-dependent correlations

$$C_{ik,t}(\alpha) = \sum_{\mathbf{s}_t,\mathbf{s}_{t-1}} (s_{i,t} - m_{i,t}(\alpha))(s_{k,t} - m_{k,t}(\alpha)) P_\alpha^{[t-1:t]}(\mathbf{s}_t, \mathbf{s}_{t-1}), \tag{48}$$

around $\alpha = 0$:

$$C_{ik,t}(\alpha) = \sum_{n=0}^{m} \frac{\alpha^n}{n!} \frac{\partial^n C_{ik,t}(\alpha = 0)}{\partial \alpha^n} + \mathcal{O}(\alpha^{(m+1)}). \tag{49}$$

The derivatives with different orders, and their values evaluated at $\alpha = 0$ are obtained as follows.

The zeroth order term is:

$$C_{ik,t}(\alpha) = \sum_{\mathbf{s}_{t-1},\mathbf{s}_{t-2}} (\tanh h_{i,t}(\alpha) - m_{i,t}(\alpha))(\tanh h_{k,t}(\alpha) - m_{k,t}(\alpha)) P_\alpha(\mathbf{s}_{t-1}|\mathbf{s}_{t-2}) P(\mathbf{s}_{t-2}), \tag{50}$$

$$C_{ik,t}(\alpha = 0) = (m_{i,t} - m_{i,t})(m_{k,t} - m_{k,t}) = 0. \tag{51}$$

The first order term is:

$$\frac{\partial C_{ik,t}(\alpha)}{\partial \alpha} = \sum_{\mathbf{s}_{t-1},\mathbf{s}_{t-2}} \Big(\big((1 - \tanh^2 h_{i,t}(\alpha))\Delta h_{i,t} - \frac{\partial m_{i,t}(\alpha)}{\partial \alpha}\big)\big(\tanh h_{k,t}(\alpha) - m_{k,t}(\alpha)\big) P_\alpha(\mathbf{s}_{t-1}|\mathbf{s}_{t-2}) \tag{52}$$

$$+ \big((1 - \tanh^2 h_{k,t}(\alpha))\Delta h_{k,t} - \frac{\partial m_{k,t}(\alpha)}{\partial \alpha}\big)\big(\tanh h_{i,t}(\alpha) - m_{i,t}(\alpha)\big) P_\alpha(\mathbf{s}_{t-1}|\mathbf{s}_{t-2}) \tag{53}$$

$$+ \sum_{m} (\tanh h_{i,t}(\alpha) - m_{i,t}(\alpha))(\tanh h_{k,t}(\alpha) - m_{k,t}(\alpha)) \frac{\partial P_\alpha(\mathbf{s}_{t-1}|\mathbf{s}_{t-2})}{\partial \alpha} \Big) P(\mathbf{s}_{t-2}). \tag{54}$$

Here, all terms are equal to zero for $\alpha = 0$ because the terms $\tanh h_{i,t}(\alpha = 0) = m_{i,t}$ cancel out. Then we have

$$\frac{\partial C_{ik,t}(\alpha = 0)}{\partial \alpha} = 0. \tag{55}$$

Therefore the nMF equation is obtained as

$$C_{ik,t} \approx 0. \tag{56}$$

The second order term is:

$$\frac{\partial^2 C_{ik,t}(\alpha)}{\partial \alpha^2} = \sum_{\mathbf{s}_{t-1},\mathbf{s}_{t-2}} \Big(2\big((1 - \tanh^2 h_{i,t}(\alpha))\Delta h_{i,t} - \frac{\partial m_{i,t}(\alpha)}{\partial \alpha}\big)\big((1 - \tanh^2 h_{k,t}(\alpha))\Delta h_{k,t} - \frac{\partial m_{k,t}(\alpha)}{\partial \alpha}\big) P_\alpha(\mathbf{s}_{t-1}|\mathbf{s}_{t-2}) \tag{57}$$

$$+ \big(-2 \tanh h_{i,t}(\alpha)(1 - \tanh^2 h_{i,t}(\alpha))\Delta h_{i,t}^2 - \frac{\partial^2 m_{i,t}(\alpha)}{\partial \alpha^2}\big)\big(\tanh h_{k,t}(\alpha) - m_{k,t}(\alpha)\big) P_\alpha(\mathbf{s}_{t-1}|\mathbf{s}_{t-2}) \tag{58}$$

$$+ \big(-2 \tanh h_{k,t}(\alpha)(1 - \tanh^2 h_{k,t}(\alpha))\Delta h_{k,t}^2 - \frac{\partial^2 m_{k,t}(\alpha)}{\partial \alpha^2}\big)\big(\tanh h_{i,t}(\alpha) - m_{i,t}(\alpha)\big) P_\alpha(\mathbf{s}_{t-1}|\mathbf{s}_{t-2}) \tag{59}$$

$$+ (\tanh h_{i,t}(\alpha) - m_{i,t}(\alpha))(\tanh h_{k,t}(\alpha) - m_{k,t}(\alpha)) \frac{\partial^2 P_\alpha(\mathbf{s}_{t-1}|\mathbf{s}_{t-2})}{\partial \alpha^2} \tag{60}$$

$$+ \big((1 - \tanh^2 h_{i,t}(\alpha))\Delta h_{i,t} - \frac{\partial m_{i,t}(\alpha)}{\partial \alpha}\big)(\tanh h_{k,t}(\alpha) - m_{k,t}(\alpha)) \frac{\partial P_\alpha(\mathbf{s}_{t-1}|\mathbf{s}_{t-2})}{\partial \alpha} \tag{61}$$

$$+ (\tanh h_{i,t}(\alpha) - m_{i,t}(\alpha))\big((1 - \tanh^2 h_{k,t}(\alpha))\Delta h_{k,t} - \frac{\partial m_{k,t}(\alpha)}{\partial \alpha}\big) \frac{\partial P_\alpha(\mathbf{s}_{t-1}|\mathbf{s}_{t-2})}{\partial \alpha} \Big) P(\mathbf{s}_{t-2}). \tag{62}$$

Except for the term in the first line, for $\alpha = 0$ all terms are equal to zero because $\tanh h_{i,t}(\alpha = 0) = m_{i,t}$ cancel out. This gives

$$\frac{\partial^2 C_{ik,t}(\alpha=0)}{\partial\alpha^2} = 2(1-m_{i,t}^2)(1-m_{k,t}^2)\sum_j J_{ij}J_{kj}(1-m_{j,t-1}^2). \tag{63}$$

Hence the correlations expanded up to the second order can be described as:

$$C_{ik,t}(\alpha) = C_{ik,t}(\alpha=0) + \alpha\frac{\partial C_{ik,t}(\alpha=0)}{\partial\alpha} + \frac{\alpha^2}{2}\frac{\partial^2 C_{ik,t}(\alpha=0)}{\partial\alpha^2} + \mathcal{O}(\alpha^3) \tag{64}$$

$$= \alpha^2(1-m_{i,t}^2)(1-m_{k,t}^2)\sum_j J_{ij}J_{kj}(1-m_{j,t-1}^2) + \mathcal{O}(\alpha^3). \tag{65}$$

Hence, the corresponding TAP approximation is obtained as

$$C_{ik,t} \approx (1-m_{i,t}^2)(1-m_{k,t}^2)\sum_j J_{ij}J_{kj}(1-m_{j,t-1}^2). \tag{66}$$

Time-delayed correlations

Similarly to the equal-time correlations, we approximate $D_{il,t}$ by evaluating the Plefka expansion of $D_{il,t}(\alpha = 1)$ around $\alpha = 0$.

We describe time-delayed correlations of the system

$$D_{il,t}(\alpha) = \sum_{\mathbf{s}_t,\mathbf{s}_{t-1}} (s_{i,t} - m_{i,t}(\alpha))(s_{l,t-1} - m_{l,t-1}(\alpha))P_\alpha^{[t-1:t]}(\mathbf{s}_t, \mathbf{s}_{t-1}), \tag{67}$$

using an expansion:

$$D_{il,t}(\alpha) = \sum_{k=0}^{n}\frac{\alpha^k}{k!}\frac{\partial^k D_{il,t}(\alpha=0)}{\partial\alpha^k} + \mathcal{O}(\alpha^{(n+1)}). \tag{68}$$

Likewise, the zeroth order term yields:

$$D_{il,t}(\alpha) = \sum_{\mathbf{s}_{t-1},\mathbf{s}_{t-2}} (\tanh h_{i,t}(\alpha) - m_{i,t}(\alpha))(s_{l,t-1} - m_{l,t-1}(\alpha))P_\alpha(\mathbf{s}_{t-1}|\mathbf{s}_{t-2})P(\mathbf{s}_{t-2}). \tag{69}$$

$$D_{il,t}(\alpha=0) = 0. \tag{70}$$

The first order term is:

$$\frac{\partial D_{il,t}(\alpha)}{\partial\alpha} = \sum_{\mathbf{s}_{t-1},\mathbf{s}_{t-2}} \Big(\big((1-\tanh^2 h_{i,t}(\alpha))\Delta h_{i,t} - \frac{\partial m_{i,t}(\alpha)}{\partial\alpha}\big)(s_{l,t-1} - m_{l,t-1}(\alpha))P_\alpha(\mathbf{s}_{t-1}|\mathbf{s}_{t-2}) \tag{71}$$

$$+ (\tanh h_{i,t}(\alpha) - m_{i,t}(\alpha))\big((s_{l,t-1} - m_{l,t-1}(\alpha))\frac{\partial P_\alpha(\mathbf{s}_{t-1}|\mathbf{s}_{t-2})}{\partial\alpha} - \frac{\partial m_{l,t-1}(\alpha)}{\partial\alpha}P_\alpha(\mathbf{s}_{t-1}|\mathbf{s}_{t-2})\big)\Big)P(\mathbf{s}_{t-2}). \tag{72}$$

When evaluated at $\alpha = 0$, the terms in the second line disappear because the terms $\tanh h_{i,t}(\alpha = 0) = m_{i,t}$ cancel out. Note that the term in the first line is computed as

$$\big((1-\tanh^2 h_{i,t}(0))\Delta h_{i,t} - \frac{\partial m_{i,t}(\alpha)}{\partial\alpha} = (1-m_{i,t}^2)\sum_j J_{ij}(s_{j,t-1} - m_{j,t-1}), \tag{73}$$

which is multiplied by $s_{l,t-1} - m_{l,t-1}(\alpha)$. Because we take expectation over $\mathbf{s}_{t-1}$ with the independent model, only expectation of the term $(s_{l,t-1} - m_{l,t-1})^2$ is preserved. Thus we have

$$\frac{\partial D_{il,t}(\alpha=0)}{\partial\alpha} = (1-m_{i,t}^2)J_{il}(1-m_{l,t-1}^2). \tag{74}$$

Therefore the nMF equation is obtained as:

$$D_{il,t} \approx J_{il}(1-m_{i,t}^2)(1-m_{l,t-1}^2). \tag{75}$$

The second order term is:

$$\frac{\partial^2 D_{il,t}(\alpha)}{\partial\alpha^2} = \sum_{\mathbf{s}_{t-1},\mathbf{s}_{t-2}} \Big(-2\tanh h_{i,t}(\alpha)(1-\tanh^2 h_{i,t}(\alpha))\Delta h_{i,t}^2 - \frac{\partial^2 m_{i,t}(\alpha)}{\partial\alpha^2}\Big)(s_{l,t-1}-m_{l,t-1}(\alpha))P_\alpha(\mathbf{s}_{t-1}|\mathbf{s}_{t-2}) \tag{76}$$

$$+2\big((1-\tanh^2 h_{i,t}(\alpha))\Delta h_{i,t} - \frac{\partial m_{i,t}(\alpha)}{\partial\alpha}\big)\big((s_{l,t-1}-m_{l,t-1}(\alpha))\frac{\partial P_\alpha(\mathbf{s}_{t-1}|\mathbf{s}_{t-2})}{\partial\alpha} \tag{77}$$

$$-\frac{\partial m_{l,t-1}(\alpha)}{\partial\alpha}P_\alpha(\mathbf{s}_{t-1}|\mathbf{s}_{t-2})\big) \tag{78}$$

$$+(\tanh h_{i,t}(\alpha)-m_{i,t}(\alpha))\Big(\big((s_{l,t-1}-m_{l,t-1}(\alpha))\frac{\partial^2 P_\alpha(\mathbf{s}_{t-1}|\mathbf{s}_{t-2})}{\partial\alpha^2} \tag{79}$$

$$-2\frac{\partial m_{l,t-1}(\alpha)}{\partial\alpha}\frac{\partial P_\alpha(\mathbf{s}_{t-1}|\mathbf{s}_{t-2})}{\partial\alpha} - \frac{\partial^2 m_{l,t-1}(\alpha)}{\partial\alpha^2}P_\alpha(\mathbf{s}_{t-1}|\mathbf{s}_{t-2})\Big)P(\mathbf{s}_{t-2}). \tag{80}$$

Here, in the first line, only the $\Delta h_{i,t}^2(s_{l,t-1}-m_{l,t-1})$ survives as

$$\Delta h_{i,t}^2(s_{l,t-1}-m_{l,t-1}) = \Big(-\Theta_{i,t}+H_i+\sum_j J_{ij}m_{j,t-1}+\sum_j J_{ij}(s_{j,t-1}-m_{j,t-1})\Big)^2(s_{l,t-1}-m_{l,t-1}) \tag{81}$$

$$= \Big(-\Theta_{i,t}+H_i+\sum_j J_{ij}m_{j,t-1}\Big)^2(s_{l,t-1}-m_{l,t-1}) \tag{82}$$

$$+2\Big(-\Theta_{i,t}+H_i+\sum_j J_{ij}m_{j,t-1}\Big)\sum_j J_{ij}(s_{j,t-1}-m_{j,t-1})(s_{l,t-1}-m_{l,t-1}) \tag{83}$$

$$+\Big(\sum_j J_{ij}(s_{j,t-1}-m_{j,t-1})\Big)^2(s_{l,t-1}-m_{l,t-1}), \tag{84}$$

which results in $2(-\Theta_{i,t}+H_i+\sum_j J_{ij}m_{j,t-1})(1-m_{l,t-1}^2) - 2J_{il}^2 m_{l,t-1}(1-m_{l,t-1}^2)$ when evaluated for the mean field model.

The term in the second and third lines of the previous equation can be decomposed in two terms. The second part results in $\sum_j J_{ij}(s_{j,t-1}-m_{j,t-1})\frac{\partial m_{l,t-1}(\alpha)}{\partial\alpha}$, which results in zero. From the first term, only survives the part containing $\sum_j J_{ij}(s_{j,t-1}-m_{j,t-1})(s_{l,t-1}-m_{l,t-1})\frac{\partial P_\alpha(\mathbf{s}_{t-1}|\mathbf{s}_{t-2})}{\partial\alpha}$ which for $\alpha=0$ results in

$$\sum_{\mathbf{s}_{t-1},\mathbf{s}_{t-2}}\sum_j J_{ij}(s_{j,t-1}-m_{j,t-1})(s_{l,t-1}-m_{l,t-1})\frac{\partial P_0(s_{t-1}|s_{t-2})}{\partial\alpha}P(s_{t-2}) \tag{85}$$

$$=\sum_{\mathbf{s}_{t-2}}\sum_{jk} J_{ij}(s_{j,t-1}-m_{j,t-1})(s_{l,t-1}-m_{l,t-1})(s_{k,t-1}-m_{k,t-1})\Delta h_{k,t-1}Q(s_{t-1})P(s_{t-2}) \tag{86}$$

$$=\sum_{\mathbf{s}_{t-1}} J_{il}(s_{l,t-1}-m_{l,t-1})^3 Q(s_{t-1})\sum_{\mathbf{s}_{t-2}}\Delta h_{k,t-1}P(s_{t-2}) \tag{87}$$

$$=-2J_{il}m_{l,t-1}(1-m_{l,t-1}^2)(-\Theta_{i,t-1}+H_i+\sum_j J_{ij}m_{j,t-2}). \tag{88}$$

The term in the last two lines disappear because the terms $\tanh h_{i,t}(\alpha=0)=m_{i,t}$ cancel out. Thus, evaluated at $\alpha=0$ we have

$$\frac{\partial^2 D_{il,t}(\alpha=0)}{\partial\alpha^2} = 4m_{i,t}(1-m_{i,t}^2)J_{il}^2 m_{l,t-1}(1-m_{l,t-1}^2) \tag{89}$$

$$-4m_{i,t}(1-m_{i,t}^2)J_{il}(1-m_{l,t-1}^2)(-\Theta_{i,t}+H_i+\sum_j m_{j,t-1}) \tag{90}$$

$$-4(1-m_{i,t}^2)J_{il}m_{l,t-1}(1-m_{l,t-1}^2)(-\Theta_{i,t-1}+H_i+\sum_j J_{ij}m_{j,t-2}). \tag{91}$$

The second and third term above are equal to $0+\mathcal{O}(\alpha)$, which makes them negligible (i.e. order larger than quadratic) when computing $\alpha^2 \frac{\partial^2 D_{il,t}(\alpha=0)}{\partial\alpha^2}$.

Hence the second order expansion is

$$D_{il,t}(\alpha) = D_{il,t}(\alpha=0) + \alpha\frac{\partial D_{il,t}(\alpha=0)}{\partial\alpha} + \frac{\alpha^2}{2}\frac{\partial^2 D_{il,t}(\alpha=0)}{\partial\alpha^2} + \mathcal{O}(\alpha^3) \quad (92)$$

$$= \alpha(1-m_{i,t}^2)J_{il}(1-m_{l,t-1}^2) + \alpha^2 2m_{i,t}(1-m_{i,t}^2)J_{il}^2 m_{l,t-1}(1-m_{l,t-1}^2) + \mathcal{O}(\alpha^3). \quad (93)$$

Thus the TAP equation for the time-delayed correlations can be described as:

$$D_{il,t} \approx J_{il}(1-m_{i,t}^2)(1-m_{l,t-1}^2)(1+2J_{il}m_{i,t}m_{l,t-1}). \quad (94)$$

Supplementary Note 3: Plefka[t]

This approximation uses the following approximated marginal probability distribution:

$$P_\alpha^{[t]}(\mathbf{s}_t) = \sum_{\mathbf{s}_{t-1}} P_\alpha(\mathbf{s}_t|\mathbf{s}_{t-1}) P(\mathbf{s}_{t-1}), \tag{95}$$

where

$$P_\alpha(s_{i,t}|\mathbf{s}_{t-1}) = \frac{\mathrm{e}^{s_{i,t} h_{i,t}(\alpha)}}{2\cosh h_{i,t}(\alpha)}, \tag{96}$$

$$h_{i,t}(\alpha) = (1-\alpha)\Theta_{i,t} + \alpha(H_i + \sum_j J_{ij} s_{j,t-1}). \tag{97}$$

We define $h_{i,t}(\alpha) = \Theta_{i,t} + \alpha\, \Delta h_{i,t}$, where $\Delta h_{i,t} = -\Theta_{i,t} + H_i + \sum_j J_{ij} s_{j,t-1}$ represents deviation from the independent model. We estimate $m_{i,t}$ by using its α-dependent approximation, whose element is defined as

$$m_{i,t}(\alpha) = \sum_{\mathbf{s}_t,\mathbf{s}_{t-1}} s_{i,t} P_\alpha^{[t]}(\mathbf{s}_t, \mathbf{s}_{t-1}) = \sum_{\mathbf{s}_{t-1}} \tanh h_{i,t}(\alpha) P(\mathbf{s}_{t-1}). \tag{98}$$

Approximating its value by expanding around $\alpha = 0$ yields

$$m_{i,t}(\alpha) = m_{i,t}(\alpha=0) + \sum_{k=1}^{n} \frac{\alpha^k}{k!} \frac{\partial^k m_{i,t}(\alpha=0)}{\partial \alpha^k} + \mathcal{O}(\alpha^{(n+1)}). \tag{99}$$

By noting that $m_{i,t}(\alpha=0) = m_{i,t}(\alpha=1)$, we evaluate it at $\alpha = 1$. This results in

$$\left[\sum_{k=1}^{n} \frac{\alpha^k}{k!} \frac{\partial^k m_{i,t}(\alpha=0)}{\partial \alpha^k}\right]_{\alpha=1} = 0 + \left[\mathcal{O}(\alpha^{(n+1)})\right]_{\alpha=1}. \tag{100}$$

The approximation yields the nMF equations when we ignore the quadratic term and higher, and the TAP equations when ignoring the third and higher order terms. The terms at each order and its evaluation at $\alpha = 0$ are obtained as follows.

The first order term is:

$$\frac{\partial m_{i,t}(\alpha)}{\partial \alpha} = \sum_{\mathbf{s}_{t-1}} \Big(1 - \tanh^2 h_{i,t}(\alpha)\Big) \Delta h_{i,t} P(\mathbf{s}_{t-1}), \tag{101}$$

$$\frac{\partial m_{i,t}(\alpha=0)}{\partial \alpha} = (1 - m_{i,t}^2)\Big(-\Theta_{i,t} + H_i + \sum_j J_{ij} m_{j,t-1}\Big). \tag{102}$$

The first order or naive mean-field approximation is then

$$\Theta_{i,t} = H_i + \sum_j J_{ij} m_{j,t-1} + \left[\mathcal{O}(\alpha^1)\right]_{\alpha=1}, \tag{103}$$

$$m_i(t) \approx \tanh\,[H_i + \sum_j J_{ij} m_{j,t-1}]. \tag{104}$$

For the second order approximation, we have

$$\frac{\partial^2 m_{i,t}(\alpha)}{\partial \alpha_t^2} = -2 \sum_{\mathbf{s}_{t-1}} \tanh h_{i,t}(\alpha) \Big(1 - \tanh^2 h_{i,t}(\alpha)\Big) \Delta h_{i,t}^2 P(\mathbf{s}_{t-1}), \tag{105}$$

$$\frac{\partial^2 m_{i,t}(\alpha=0)}{\partial \alpha_t^2} = -2 m_{i,t}\Big(1 - m_{i,t}^2\Big) \Bigg[\Big(-\Theta_{i,t} + H_i + \sum_j J_{ij} m_{j,t-1}\Big)^2 \tag{106}$$

$$+ \sum_{j,k} J_{ij} J_{ik} C_{jk,t-1}\Bigg]. \tag{107}$$

Then we solve the following second order equation:

$$\frac{\partial m_{i,t}(\alpha=0)}{\partial\alpha} + \frac{1}{2}\frac{\partial^2 m_{i,t}(\alpha=0)}{\partial\alpha^2} = 0 + \mathcal{O}(\alpha^3)|_{\alpha=1}. \tag{108}$$

To compute the second order approximation, we can take advantage of the fact that the second order term contains the expression $\Big[\alpha^2(-\Theta_{i,t}+H_i+\sum_j J_{ij}m_j)^2\Big]_{\alpha=1}$. We know that $\Big[\alpha(1-m_i^2)(-\Theta_{i,t}+H_i+\sum_j J_{ij}m_j)\Big]_{\alpha=1} = \big[\mathcal{O}(\alpha^2)\big]_{\alpha=1}$ and thus $\Big[\alpha^2(-\Theta_{i,t}+H_i+\sum_j J_{ij}m_j)^2\Big]_{\alpha=1} = \big[\mathcal{O}(\alpha^4)\big]_{\alpha=1}$. This yields the TAP equations:

$$\Theta_{i,t} = H_i + \sum_j J_{ij}m_{j,t-1} - m_{i,t}\sum_{j,k} J_{ij}J_{ik}C_{jk,t-1} + \big[\mathcal{O}(\alpha^2)\big]_{\alpha=1}, \tag{109}$$

$$m_i(t) \approx \tanh[H_i + \sum_j J_{ij}m_{j,t-1} - m_{i,t}\sum_{j,k} J_{ij}J_{ik}C_{jk,t-1}]. \tag{110}$$

This result is a reminiscence of the TAP equations obtained for symmetric networks and asymmetric networks, which generally take the form $\Theta_{i,t} \approx H_i + \sum_j J_{ij}m_{j,t-1} - m_{i,t}\sum_j J_{ij}^2(1-m_j^2)$ [1, 2]. The only difference is that previous results approximated either the stationary state of the network, or the probability of trajectories over several updates of the network dynamics. The consequence of these is that previous results ignored correlations at previous states (since they were also expanded from the independent model, thus C_{jl} terms become zero when $i \neq j$ and $1-m_i^2$ otherwise).

Equal-time correlations

When $i=j$, $C_{ii,t} = 1 - m_{i,t}(\alpha)^2$. When $i \neq j$, correlations in the system can be obtained by expanding

$$C_{ik,t}(\alpha) = \sum_{\mathbf{s}_t,\mathbf{s}_{t-1}} (s_{i,t} - m_{i,t}(\alpha))(s_{k,t} - m_{k,t}(\alpha))P_\alpha(\mathbf{s}_t|\mathbf{s}_{t-1})P(\mathbf{s}_{t-1}), \tag{111}$$

over $\alpha = 0$:

$$C_{ik,t}(\alpha) = \sum_{n=0}^{m} \frac{\alpha^n}{n!}\frac{\partial^n C_{ik,t}(\alpha=0)}{\partial\alpha^n} + \mathcal{O}(\alpha^{(m+1)}). \tag{112}$$

The expanded terms of each order and its evaluation at $\alpha = 0$ are obtained as follows.
The zeroth order term is:

$$C_{ik,t}(\alpha) = \sum_{\mathbf{s}_{t-1}} (\tanh h_{i,t}(\alpha) - m_{i,t}(\alpha))(\tanh h_{k,t}(\alpha) - m_{k,t}(\alpha))P(\mathbf{s}_{t-1}), \tag{113}$$

$$C_{ik,t}(\alpha=0) = (m_{i,t} - m_{i,t})(m_{k,t} - m_{k,t}) = 0. \tag{114}$$

The first order term yields

$$\frac{\partial C_{ik,t}(\alpha)}{\partial\alpha} = \sum_{\mathbf{s}_{t-1}} \Big(\big((1-\tanh^2 h_{i,t}(\alpha))\Delta h_{i,t} \tag{115}$$

$$- \frac{\partial m_{i,t}(\alpha)}{\partial\alpha}\big)\big(\tanh h_{i,t}(\alpha) - m_{k,t}(\alpha)\big) \tag{116}$$

$$+ \big((1-\tanh^2 h_{k,t}(\alpha))\Delta h_{k,t} \tag{117}$$

$$- \frac{\partial m_{k,t}(\alpha)}{\partial\alpha}\big)\big(\tanh h_{i,t}(\alpha) - m_{i,t}(\alpha)\big)\Big)P(\mathbf{s}_{t-1}), \tag{118}$$

$$\frac{\partial C_{ik,t}(\alpha=0)}{\partial\alpha} = 0. \tag{119}$$

Therefore the nMF equation is obtained as

$$C_{ik,t} \approx 0. \tag{120}$$

The second order term is:

$$\frac{\partial^2 C_{ik,t}(\alpha)}{\partial\alpha^2} = \sum_{\mathbf{s}_{t-1}} \Big(2\big((1-\tanh^2 h_{i,t}(\alpha))\Delta h_{i,t} \tag{121}$$

$$- \frac{\partial m_{i,t}(\alpha)}{\partial\alpha}\big)\big((1-\tanh^2 h_{k,t}(\alpha))\Delta h_{k,t} - \frac{\partial m_{k,t}(\alpha)}{\partial\alpha}\big) \tag{122}$$

$$+ \big(-2\tanh h_{i,t}(\alpha)(1-\tanh^2 h_{i,t}(\alpha))\Delta h_{i,t}^2 \tag{123}$$

$$- \frac{\partial^2 m_{i,t}(\alpha)}{\partial\alpha^2}\big)\big(\tanh h_{k,t}(\alpha) - m_{k,t}(\alpha)\big) \tag{124}$$

$$+ \big(-2\tanh h_{k,t}(\alpha)(1-\tanh^2 h_{k,t}(\alpha))\Delta h_{k,t}^2 \tag{125}$$

$$- \frac{\partial^2 m_{k,t}(\alpha)}{\partial\alpha^2}\big)\big(\tanh h_{i,t}(\alpha) - m_{i,t}(\alpha)\big)\Big) P(\mathbf{s}_{t-1}), \tag{126}$$

$$\frac{\partial^2 C_{ik,t}(\alpha=0)}{\partial\alpha^2} = 2(1-m_{i,t}^2)(1-m_{k,t}^2)(\sum_{ln} J_{il} J_{jn} C_{ln,t-1}). \tag{127}$$

So the TAP equation for the correlations can be described as:

$$C_{ik,t} \approx (1-m_{i,t}^2)(1-m_{k,t-1}^2) \sum_{ln} J_{il} J_{jn} C_{ln,t-1}. \tag{128}$$

To obtain this result, correlations of order 3 (and more) $C_{ijk,t} = \sum_{\mathbf{s}_t}(s_{i,t}-m_{i,t})(s_{j,t}-m_{j,t})(s_{k,t}-m_{k,t})P(\mathbf{s}_t) = 0 + \big[\mathcal{O}(\alpha^3)\big]_{\alpha=1}$ are ignored in the TAP equations if $i \neq j \neq k$. Otherwise we used $C_{iik,t} = -2m_{i,t}C_{ik,t}$ and $C_{iii,t} = -2m_{i,t}(1-m_{i,t}^2)$.

The obtained expression has a form that is similar to the equations in [3] but presents some differences since is computed from the same expansion as the TAP equations, instead of performing a new expansion from the approximation of $\Theta_{i,t}$.

Time-delayed correlations

We introduce the α-dependent time-delayed correlations as

$$D_{il,t}(\alpha) = \sum_{\mathbf{s}_t,\mathbf{s}_{t-1}} (s_{i,t} - m_{i,t}(\alpha))(s_{l,t-1} - m_{l,t-1}) P_\alpha(\mathbf{s}_t|\mathbf{s}_{t-1}) P(\mathbf{s}_{t-1}). \tag{129}$$

We approximate $D_{il,t}$ by expanding this equation around $\alpha = 0$.

The zeroth order term yields:

$$D_{il,t}(\alpha) = \sum_{\mathbf{s}_{t-1}} (\tanh h_{i,t}(\alpha) - m_{i,t}(\alpha))(s_{l,t-1} - m_{l,t-1}) P(\mathbf{s}_{t-1}), \tag{130}$$

$$D_{il,t}(\alpha=0) = 0. \tag{131}$$

The first order term is:

$$\frac{\partial D_{il,t}(\alpha)}{\partial\alpha} = \sum_{\mathbf{s}_{t-1}} \sum_{\mathbf{s}_{t-1}} \Big(\big((1-\tanh^2 h_{i,t}(\alpha))\Delta h_{i,t} \tag{132}$$

$$- \frac{\partial m_{i,t}(\alpha)}{\partial\alpha}\big)(s_{l,t-1} - m_{l,t-1})\Big) P(\mathbf{s}_{t-1}), \tag{133}$$

$$\frac{\partial D_{il,t}(\alpha=0)}{\partial\alpha} = (1-m_{i,t}^2) \sum_j J_{ij} C_{jl,t-1}, \tag{134}$$

therefore obtaining that for the nMF equation:

$$D_{il,t} \approx (1-m_{i,t}^2) \sum_j J_{ij} C_{jl,t-1}. \tag{135}$$

The second order term is:

$$\frac{\partial^2 D_{il,t}(\alpha)}{\partial\alpha^2} = \sum_{\mathbf{s}_{t-1}} \Big(\big(-2\tanh h_{i,t}(\alpha)(1-\tanh^2 h_{i,t}(\alpha))\Delta h_{i,t}^2 \tag{136}$$

$$- \frac{\partial^2 m_{i,t}(\alpha)}{\partial\alpha^2}\big)(s_{l,t-1} - m_{l,t-1})\Big) P(\mathbf{s}_{t-1}) \tag{137}$$

$$= -2\tanh h_{i,t}(\alpha)(1-\tanh^2 h_{i,t}(\alpha)) \sum_{\mathbf{s}_{t-1}} \Delta h_{i,t}^2 (s_{l,t-1} - m_{l,t-1}) P(\mathbf{s}_{t-1}). \tag{138}$$

Here we note that

$$\sum_{\mathbf{s}_{t-1}} \Delta h_{i,t}^2 (s_{l,t-1} - m_{l,t-1}) P(\mathbf{s}_{t-1}) \tag{139}$$

$$= \sum_{\mathbf{s}_{t-1}} \Big(-\Theta_{i,t} + H_i + \sum_j J_{ij} m_{j,t-1} + \sum_j J_{ij}(s_{j,t-1} - m_{j,t-1}) \Big)^2 (s_{l,t-1} - m_{l,t-1}) P(\mathbf{s}_{t-1}) \tag{140}$$

$$= \Big(-\Theta_{i,t} + H_i + \sum_j J_{ij} m_{j,t-1} \Big)^2 \sum_{\mathbf{s}_{t-1}} (s_{l,t-1} - m_{l,t-1}) P(\mathbf{s}_{t-1}) \tag{141}$$

$$+ 2\Big(-\Theta_{i,t} + H_i + \sum_j J_{ij} m_{j,t-1} \Big) \sum_{\mathbf{s}_{t-1}} \sum_j J_{ij}(s_{j,t-1} - m_{j,t-1})(s_{l,t-1} - m_{l,t-1}) P(\mathbf{s}_{t-1}) \tag{142}$$

$$+ \sum_{\mathbf{s}_{t-1}} \Big(\sum_j J_{ij}(s_{j,t-1} - m_{j,t-1}) \Big)^2 (s_{l,t-1} - m_{l,t-1}) P(\mathbf{s}_{t-1}) \tag{143}$$

$$= 2\Big(-\Theta_{i,t} + H_i + \sum_j J_{ij} m_{j,t-1} \Big) \sum_j J_{ij} C_{jl,t-1} + \sum_{j,k} J_{ij} J_{ik} C_{jkl,t-1}. \tag{144}$$

where $C_{jkl,t-1} \equiv \sum_{\mathbf{s}_{t-1}} (s_{j,t-1} - m_{j,t-1})(s_{k,t-1} - m_{k,t-1})(s_{l,t-1} - m_{l,t-1}) P(\mathbf{s}_{t-1})$. We then obtain

$$\frac{\partial^2 D_{il,t}(\alpha=0)}{\partial\alpha^2} = -2m_{i,t}(1-m_{i,t}^2) \sum_{j,k} J_{ij} J_{ik} C_{jkl,t-1} - 4m_{i,t}(1-m_{i,t}^2)(-\Theta_{i,t} + H_i + \sum_j J_{ij} m_j) \sum_j J_{ij} C_{jl,t-1}. \tag{145}$$

Here, the second term above is equal to $0 + \mathcal{O}(\alpha)$, which makes it negligible (i.e. order larger than quadratic) when computing $\alpha^2 \frac{\partial^2 D_{il,t}(\alpha=0)}{\partial\alpha^2}$. Thus the second order expansion for delayed correlations can be described as:

$$D_{il,t} = (1-m_{i,t}^2)\big(\sum_j J_{ij} C_{jl,t-1} - m_{i,t} \sum_{j,k} J_{ij} J_{ik} C_{jkl,t-1} \big) + \big[\mathcal{O}(\alpha^3)\big]_{\alpha=1}. \tag{146}$$

Moreover, if we consider that $C_{jkl,t-1}$ terms are going to be equal to $0 + \big[\mathcal{O}(\alpha^3)\big]_{\alpha=1}$ when $j \neq l \neq n$, we have the TAP approximation

$$D_{il,t} \approx (1-m_{i,t}^2) \sum_j J_{ij} C_{jl,t-1} (1 + 2J_{il} m_{i,t} m_{l,t-1}) + \big[\mathcal{O}(\alpha^3)\big]_{\alpha=1}. \tag{147}$$

The nMF Equation for time-delayed correlations is similar to the first order approximation obtained by [3]. The second order approximation differs since they do a new expansion over one time step of the obtained TAP expression obtained for the whole trajectory. In our case, since we apply the TAP expansion for one-step updates in all cases, we can derive an expression from the same expansion that obtains the TAP equation for updating the mean fields of the system.

Supplementary Note 4: Plefka[$t-1$]

This approximation uses the following approximated marginal probability distribution.

$$P^{[t-1]}_{\alpha}(\mathbf{s}_t, \mathbf{s}_{t-1}) = \sum_{\mathbf{s}_{t-2}} P(\mathbf{s}_t|\mathbf{s}_{t-1}) P_{\alpha}(\mathbf{s}_{t-1}|\mathbf{s}_{t-2}) P(\mathbf{s}_{t-2}), \tag{148}$$

where activity at time t is normally defined by

$$P(s_{i,t}|\mathbf{s}_{t-1}) = \frac{\mathrm{e}^{s_{i,t}h_{i,t}}}{2\cosh h_{i,t}}, \tag{149}$$

$$h_{i,t} = H_i + \sum_j J_{ij} s_{j,t-1}, \tag{150}$$

whereas activity at time $t-1$ is mediated by the parameter α

$$P_{\alpha}(s_{i,t-1}|\mathbf{s}_{t-2}) = \frac{\mathrm{e}^{s_{i,t-1}h_{i,t-1}(\alpha)}}{2\cosh h_{i,t-1}(\alpha)}, \tag{151}$$

$$h_{i,t-1}(\alpha) = (1-\alpha)\Theta_{i,t-1} + \alpha(H_i + \sum_j J_{ij} s_{j,t-2}). \tag{152}$$

Computing the values of $\mathbf{\Theta}_{t-1}$ is equivalent to the calculations in 3. However, in the calculations below we will see that we will need only $m_{i,t-1}$ to compute the statistics at time t. Namely, $m_{i,t-1}$ is the only value we need to know to make the approximation at $t-1$.

Now, we calculate $m_{i,t}$ by using its α-dependent approximation, defined as

$$m_{i,t}(\alpha) = \sum_{\mathbf{s}_t,\mathbf{s}_{t-1}} s_{i,t} P^{[t-1]}_{\alpha}(\mathbf{s}_t, \mathbf{s}_{t-1}) = \sum_{\mathbf{s}_{t-1},\mathbf{s}_{t-2}} \tanh h_{i,t} P_{\alpha}(\mathbf{s}_{t-1}|\mathbf{s}_{t-2}) P(\mathbf{s}_{t-2}). \tag{153}$$

Approximating its value by expanding around $\alpha = 0$ yields

$$m_{i,t}(\alpha) = m_{i,t}(\alpha=0) + \sum_{k=1}^{n} \frac{\alpha^k}{k!} \frac{\partial^k m_{i,t}(\alpha=0)}{\partial \alpha^k} + \mathcal{O}(\alpha^{(n+1)}). \tag{154}$$

We solve this equation at $\alpha = 1$. The approximation yields the nMF equations when we ignore quadratic terms and higher, and the TAP equations when ignoring third and higher order terms. However, in this case we will only compute the terms in the nMF equation, since the second order yields marginals that are complicated to evaluate. In the case of the first order term, we will show how to estimate the corresponding marginals at the end of this Supplementary Note.

The derivatives of this distribution with respect to α is given by

$$\frac{\partial P_{\alpha}(s_{i,t-1}|\mathbf{s}_{t-2})}{\partial \alpha} = (s_{i,t-1} - \tanh h_{i,t-1}(\alpha)) \Delta h_{i,t-1} P_{\alpha}(s_{i,t-1}|\mathbf{s}_{t-2}). \tag{155}$$

Again, we define $h_{i,t-1}(\alpha) = \Theta_{i,t-1} + \alpha\, \Delta h_{i,t-1}$, where $\Delta h_{i,t-1} = -\Theta_{i,t-1} + H_i + \sum_j J_{ij} s_{j,t-2}$ represents deviation from the independent model. The derivatives of each order are derived as follows.

The zeroth order term is:

$$m_{i,t}(\alpha) = \sum_{\mathbf{s}_{t-1},\mathbf{s}_{t-2}} \tanh h_{i,t} P_{\alpha}(\mathbf{s}_{t-1}|\mathbf{s}_{t-2}) P(\mathbf{s}_{t-2}), \tag{156}$$

$$m_{i,t}(\alpha=0) = \sum_{\mathbf{s}_{t-1}} \tanh h_{i,t} Q(\mathbf{s}_{t-1}). \tag{157}$$

The first order terms are:

$$\frac{\partial m_{i,t}(\alpha)}{\partial \alpha} = \sum_{\mathbf{s}_{t-1},\mathbf{s}_{t-2}} \tanh h_{i,t} \sum_k (s_{k,t-1} - \tanh h_{k,t-1}(\alpha)) \Delta h_{k,t-1} P_{\alpha}(\mathbf{s}_{t-1}|\mathbf{s}_{t-2}) P(\mathbf{s}_{t-2}), \tag{158}$$

$$\frac{\partial m_{i,t}(\alpha=0)}{\partial \alpha} = \sum_k (-\Theta_{k,t-1} + H_k + \sum_l J_{kl} m_{l,t-2}) \sum_{\mathbf{s}_{t-1}} \tanh h_{i,t} (s_{k,t-1} - m_{k,t-1}) Q(\mathbf{s}_{t-1}). \tag{159}$$

Since $[\alpha(H_k - \Theta_{k,t-1} + \sum_l J_{kl} m_{l,t-2})]_{\alpha=1} = 0 + \left[\mathcal{O}(\alpha^2)\right]_{\alpha=1}$ (3) we can ignore the first order terms, leading to the first order expansion

$$m_{i,t} \approx \Gamma_{i,t} + \left[\mathcal{O}(\alpha^2)\right]_{\alpha=1}, \tag{160}$$

where $\Gamma_{i,t} = \sum_{\mathbf{s}_{t-1}} \tanh h_{i,t} Q(\mathbf{s}_{t-1})$. Moreover, since $Q(\mathbf{s}_{t-1})$ is an independent distribution, for a large number of units, applying the central limit theorem, it can be approximated by a Gaussian distribution (see last section of this Supplementary Note).

Equal-time correlations

When $i \neq k$, correlations in the system are calculated as

$$C_{ik,t}(\alpha) = \sum_{\mathbf{s}_t, \mathbf{s}_{t-1}} (s_{i,t} - m_{i,t}(\alpha))(s_{k,t} - m_{k,t}(\alpha)) P_\alpha(\mathbf{s}_{t-1}|\mathbf{s}_{t-2})\mathbf{s}_t, \mathbf{s}_{t-1}(\mathbf{s}_t, \mathbf{s}_{t-1}). \tag{161}$$

Again, we compute this using a Plefka expansion:

$$C_{ik,t}(\alpha) = \sum_{n=0}^{m} \frac{\alpha^n}{n!} \frac{\partial^n C_{ik,t}(\alpha=0)}{\partial \alpha^n} + \mathcal{O}(\alpha^{(m+1)}). \tag{162}$$

Otherwise, when $i = j$, we have $C_{ii,t} = 1 - m_{i,t}^2$. The derivatives of each order are derived as follows.

The zeroth order term:

$$C_{ik,t}(\alpha) = \sum_{\mathbf{s}_{t-1}, \mathbf{s}_{t-2}} (\tanh h_{i,t} - m_{i,t}(\alpha))(\tanh h_{k,t} - m_{k,t}(\alpha)) P_\alpha(\mathbf{s}_{t-1}|\mathbf{s}_{t-2}) P(\mathbf{s}_{t-2}), \tag{163}$$

$$C_{ik,t}(\alpha=0) = \sum_{\mathbf{s}_{t-1}} \tanh h_{i,t} \tanh h_{k,t} Q(\mathbf{s}_{t-1}) - m_{i,t} m_{k,t} \equiv \Gamma_{ik,t}. \tag{164}$$

The first order term yields:

$$\frac{\partial C_{ik,t}(\alpha)}{\partial \alpha} = \sum_{s(t-1)} \Big((\tanh h_{i,t} - m_{i,t}(\alpha))(\tanh h_{k,t} - m_{k,t}(\alpha)) \sum_m (s_{m,t-1} - \tanh h_{m,t-1}(\alpha)) \Delta h_{m,t-1} \tag{165}$$

$$- \frac{\partial m_{i,t}(\alpha)}{\partial \alpha} (\tanh h_{k,t} - m_{k,t}(\alpha)) - (\tanh h_{i,t} - m_{i,t}(\alpha)) \frac{\partial m_{k,t}(\alpha)}{\partial \alpha} \Big) P_\alpha(\mathbf{s}_{t-1}|\mathbf{s}_{t-2}) P(\mathbf{s}_{t-2}), \tag{166}$$

$$\frac{\partial C_{ik,t}(\alpha=0)}{\partial \alpha} = - \sum_m (H_m - \Theta_{m,t-1} + \sum_n J_{mn} m_{n,t-2}) (\sum_{\mathbf{s}_{t-1}} (\tanh h_{k,t} - m_{k,t} \tag{167}$$

$$+ \tanh h_{i,t} - m_{i,t})(s_{m,t-1} - m_{m,t-1}) Q(\mathbf{s}_{t-1}). \tag{168}$$

Since $[\alpha(H_m - \Theta_{m,t-1} + \sum_n J_{mn} m_{n,t-2})]_{\alpha=1} = 0 + \left[\mathcal{O}(\alpha^2)\right]_{\alpha=1}$ (3), the nMF equation is:

$$C_{ik,t} \approx \Gamma_{ik,t}. \tag{169}$$

Time-delayed correlations

Time-delayed correlations in the system are calculated as

$$D_{il,t}(\alpha) = \sum_{\mathbf{s}_t, \mathbf{s}_{t-1}} (s_{i,t} - m_{i,t}(\alpha))(s_{l,t-1} - m_{l,t-1}(\alpha)) P_\alpha^{[t-1]}(\mathbf{s}_t, \mathbf{s}_{t-1}). \tag{170}$$

We compute this using a Plefka expansion:

$$D_{il,t}(\alpha) = \sum_{n=0}^{m} \frac{\alpha^n}{n!} \frac{\partial^n D_{il,t}(\alpha=0)}{\partial \alpha^n} + \mathcal{O}(\alpha^{(m+1)}). \tag{171}$$

The zeroth order terms are:

$$D_{il,t}(\alpha) = \sum_{\mathbf{s}_{t-1},\mathbf{s}_{t-2}} (\tanh h_{i,t} - m_{i,t}(\alpha))(s_{l,t-1} - m_{l,t-1}(\alpha))P_\alpha(\mathbf{s}_{t-1}|\mathbf{s}_{t-2})P(\mathbf{s}_{t-2}), \tag{172}$$

$$D_{il,t}(\alpha = 0) = \sum_{\mathbf{s}_{t-1}} (\tanh h_{i,t} - m_{i,t})(s_{l,t-1} - m_{l,t-1})Q(\mathbf{s}_{t-1}) \equiv \Gamma^{(l)}_{i,t}. \tag{173}$$

The first order terms are:

$$\frac{\partial D_{il,t}(\alpha)}{\partial \alpha} = \sum_{\mathbf{s}_{t-1},\mathbf{s}_{t-2}} \Big((\tanh h_{i,t} - m_{i,t}(\alpha))(s_{l,t-1} - m_{l,t-1}(\alpha)) \sum_k (s_{k,t-1} - \tanh h_{k,t-1}(\alpha))\Delta h_{k,t-1} \tag{174}$$

$$- \frac{\partial m_{i,t}(\alpha))}{\partial \alpha}(s_{l,t-1} - m_{l,t-1}(\alpha)) - (\tanh h_{i,t} - m_{i,t}(\alpha))\frac{\partial m_{l,t-1}(\alpha)}{\partial \alpha} \Big) P_\alpha(\mathbf{s}_{t-1}|\mathbf{s}_{t-2})P(\mathbf{s}_{t-2}), \tag{175}$$

$$\frac{\partial D_{il,t}(\alpha = 0)}{\partial \alpha} = \sum_k \sum_{\mathbf{s}_{t-1}} (\tanh h_{i,t} - m_{i,t})(s_{l,t-1} - m_{l,t-1}(\alpha))(s_{k,t-1} \tag{176}$$

$$- m_{k,t-1}(\alpha))(-\Theta_{k,t-1} + H_k + \sum_n J_{kn} m_{n,t-2})Q(\mathbf{s}_{t-1}). \tag{177}$$

Since $[\alpha(H_k - \Theta_{k,t-1} + \sum_n J_{kn} m_{n,t-2})]_{\alpha=1} = 0 + \left[\mathcal{O}(\alpha^2)\right]_{\alpha=1}$ (3), the nMF equation is:

$$D_{il,t} \approx \Gamma^{(l)}_{i,t}. \tag{178}$$

Gaussian approximations

The integrals for computing the first order means and correlations can be directly obtained applying the central limit theorem to approximate a set of independent binary signals to a Gaussian distribution.

Thus we obtain

$$\Gamma_{i,t} = \sum_{\mathbf{s}_{t-1}} \tanh h_{i,t} Q(\mathbf{s}_{t-1}) \approx \int \mathrm{D}_x \tanh[g_{i,t} + x\sqrt{\Delta_{i,t}}], \tag{179}$$

where D_x denotes an integral using a Gaussian distribution:

$$\mathrm{D}_x = \frac{\mathrm{d}x}{\sqrt{2\pi}} \exp\left(-\frac{1}{2}x^2\right). \tag{180}$$

The other parameters are $g_{i,t} = H_i + \sum_j J_{ij} m_{j,t-1}$ and $\Delta_{i,t} = \mathrm{Var}[h_{i,t}]_{\alpha=0} = \sum_j J_{ij}^2 (1 - m_{j,t-1}^2)$.

Similarly, we have

$$\Gamma_{ik,t} = \sum_{\mathbf{s}_{t-1}} \tanh h_{i,t} \tanh h_{k,t} Q(\mathbf{s}_{t-1}) \tag{181}$$

$$\approx \int \mathrm{D}^{\rho_{ik}}_{xy} \tanh[g_{i,t} + x\sqrt{\Delta_{i,t}}] \tanh[g_{k,t} + y\sqrt{\Delta_{k,t}}] \tag{182}$$

$$= \int \mathrm{D}_x \mathrm{D}_y \tanh[g_{i,t} + (x\sqrt{\frac{1+\rho_{ik}}{2}} + y\sqrt{\frac{1-\rho_{ik}}{2}})\sqrt{\Delta_{i,t}}] \tanh[g_{k,t} + (x\sqrt{\frac{1+\rho_{ik}}{2}} - y\sqrt{\frac{1-\rho_{ik}}{2}})\sqrt{\Delta_{k,t}}], \tag{183}$$

where $\mathrm{D}^{\rho_{ik}}_{xy}$ denotes an integral using a bivariate Gaussian distribution:

$$\mathrm{D}^{\rho_{ik}}_{xy} = \frac{\mathrm{d}x\mathrm{d}y}{2\pi\sqrt{1-\rho_{ik}^2}} \exp\left(-\frac{1}{2}\frac{(x^2+y^2) - 2\rho_{ik}xy}{1-\rho_{ik}^2}\right). \tag{184}$$

The other parameters are $\rho_{ij} = \frac{\Delta_{ij,t}}{\sqrt{\Delta_{ii,t}\Delta_{kk,t}}}$, with $\Delta_{ik,t} = \mathrm{Cov}(h_{i,t}, h_{k,t})_{\alpha=0} = \sum_j J_{ij} J_{kj}(1 - m_{j,t-1}^2)$. In the following step, we applied an orthogonal transformation to the previous bivariate distribution, involving a change of variables $x' = x\sqrt{\frac{1+\rho_{ik}}{2}} + y\sqrt{\frac{1-\rho_{ik}}{2}}$ and $y' = x\sqrt{\frac{1+\rho_{ik}}{2}} - y\sqrt{\frac{1-\rho_{ik}}{2}}$ that removes the coupling terms in the bivariate normal distribution.

The integrals of the Gaussian approximations of $\Gamma_{i,t}$ and $\Gamma_{ik,t}$ are easy to compute. The problem arises when we deal with the more complex terms $\Gamma^{(l)}_{i,t}$ in Supplementary Eq. 178, which can be computed by similar approximations but multiplies the number of integrals to be solved.

For large system sizes, terms obtained from the first and second order expressions like $\Gamma^{(l)}_{i,t}$ (Supplementary Eq. 170) can be obtained by assuming that the values of individual weights are small (e.g. $J_{ij} = \mathcal{O}(1/N)$). We can compute

$$D_{il,t} \approx \Gamma^{(l)}_{i,t} = \sum_{\mathbf{s}_{t-1}} (s_{l,t} - m_{l,t}) \tanh h_{i,t} Q(\mathbf{s}_{t-1}), \tag{185}$$

where the constant term $m_{i,t}$ was removed from Supplementary Eq. 170 as it yields zero when averaging its product with $(s_{l,t} - m_{l,t})$.

With independent spins at $t-1$, we can approximate this quantity with the aid of a bivariate Gaussin distribution of fields $h_{k,t}, h_{i,t}$. Knowing that $h_{k,t} - \langle h_{k,t}\rangle = \sum_l J_{kl}(s_{l,t-1} - m_{l,t-1})$, we can approximate the following quantity

$$\sum_l J_{kl} D_{il,t} \approx \sum_{\mathbf{s}_{t-1}} \sum_l J_{kl}(s_{l,t-1} - m_{l,t-1}) \tanh h_{i,t} Q(\mathbf{s}_{t-1}) \tag{186}$$

$$= \sum_{\mathbf{s}_{t-1}} (h_{k,t} - \langle h_{k,t}\rangle) \tanh h_{i,t} Q(\mathbf{s}_{t-1}) \tag{187}$$

$$\approx \int \mathrm{D}^{\rho_{ik}}_{xy} y\sqrt{\Delta_{k,t}} \tanh[g_{i,t} + x\sqrt{\Delta_{i,t}}]. \tag{188}$$

where $\langle h_{k,t}\rangle = H_k + \sum_l m_{l,t-1}$.

As in [4], if we assume that ρ_{ik} is small,$\mathrm{D}^{\rho_{ik}}_{xy}$ can be approximated as:

$$\mathrm{D}^{\rho_{ik}}_{xy} \approx \mathrm{D}_x \mathrm{D}_y (1 + \rho_{ik} xy). \tag{189}$$

Then

$$\sum_l J_{kl} D_{il,t} \approx \int \mathrm{D}^{\rho_{ik}}_{xy} y\sqrt{\Delta_{j,t}} \tanh[g_{i,t} + x\sqrt{\Delta_{i,t}}] \tag{190}$$

$$\approx \int \mathrm{D}_x \mathrm{D}_y (y + \rho_{ik} xy^2)\sqrt{\Delta_{k,t}} \tanh[g_{i,t} + x\sqrt{\Delta_{i,t}}] \tag{191}$$

$$= \rho_{ik}\sqrt{\Delta_{k,t}} \int D_x x \tanh[g_{i,t} + x\sqrt{\Delta_{i,t}}] \tag{192}$$

$$= \rho_{ik}\sqrt{\Delta_{i,t}\Delta_{k,t}} \int D_x \left(1 - \tanh^2[g_{i,t} + x\sqrt{\Delta_{i,t}}]\right), \tag{193}$$

where the last step was obtained by partial integration.

As $\rho_{ik}\sqrt{\Delta_{i,t}\Delta_{k,t}} = \sum_{jl} J_{ij} J_{kl} C_{jl,t-1}$ we have that

$$\sum_l J_{kl} D_{il,t} \approx a_{i,t} \sum_{jl} J_{ij} J_{kl} C_{jl,t-1}, \tag{194}$$

where

$$a_{i,t} = \int D_x \left(1 - \tanh^2[g_{i,t} + x\sqrt{\Delta_{i,t}}]\right). \tag{195}$$

Therefore, we have the following approximation for the delayed correlations,

$$D_{il,t} \approx a_{i,t} \sum_j J_{ij} C_{jl,t-1}. \tag{196}$$

Supplementary Note 5: Pairwise Plefka expansions, Plefka2[t]

Pairwise delayed spin correlations

Instead of a manifold of independent distributions, in this approximation we consider a manifold $\mathcal{Q}$ with a pairwise probability distribution:

$$Q(s_{i,t}, s_{l,t-1}) = Q(s_{i,t}|s_{l,t-1})Q(s_{l,t-1}) = \frac{\mathrm{e}^{s_{i,t}\theta_{i,t}(s_{l,t-1})}}{2\cosh\theta_{i,t}(s_{l,t-1})}\frac{\mathrm{e}^{s_{l,t-1}\Theta_{l,t-1}}}{2\cosh\Theta_{l,t-1}}, \tag{197}$$

where $\theta_{i,t}(s_{l,t-1}) = \Theta_{i,t} + \Delta_{il,t}s_{l,t-1}$. Here $Q(s_{l,t-1})$ is the independent probability distribution for $s_{l,t-1}$ computed as in 3, and $Q(s_{i,t}|s_{l,t-1})$ is a conditional probability distribution we use to construct the pairwise probability distribution $Q(s_{i,t}, s_{l,t-1})$ using the chain rule.

We use this pairwise model to approximate the distribution

$$P(s_{i,t}, s_{l,t-1}) = \sum_{\mathbf{s}_{\backslash l,t-1}} P(s_{i,t}, \mathbf{s}_{t-1}) = \sum_{\mathbf{s}_{\backslash l,t-1}} P(s_{i,t}|\mathbf{s}_{t-1})P(\mathbf{s}_{t-1}). \tag{198}$$

with $s_{\backslash l,t-1}$ containing all elements of $\mathbf{s}_{t-1}$ except $s_{l,t-1}$.

As in previous cases, we want to find an approximation of the probability distribution at time t that minimizes the relative entropy

$$D(P(s_{i,t}, s_{l,t-1})||Q(s_{i,t}, s_{l,t-1})) = \sum_{\substack{s_{i,t}\\ s_{l,t-1}}} P(s_{i,t}, s_{l,t-1}) \log\frac{P(s_{i,t}, s_{l,t-1})}{Q(s_{i,t}, s_{l,t-1})}. \tag{199}$$

Specifically, the mean-field approximation that minimizes the relative entropy is the one that satisfies

$$\frac{\partial D(P(s_{i,t}, s_{l,t-1})||Q(s_{i,t}, s_{l,t-1}))}{\partial\Theta_{i,t}} = -\sum_{\substack{s_{i,t}\\ s_{l,t-1}}} \Big(s_{i,t} - \tanh[\theta_{i,t}(s_{l,t-1})]\Big)P(s_{i,t}, s_{l,t-1}) \tag{200}$$

$$= \langle s_{i,t}\rangle_Q - \langle s_{i,t}\rangle_P = 0, \tag{201}$$

$$\frac{\partial D(P(s_{i,t}, s_{l,t-1})||Q(s_{i,t}, s_{l,t-1}))}{\partial\Delta_{il,t}} = -\sum_{\substack{s_{i,t}\\ s_{l,t-1}}} \Big(s_{i,t}s_{l,t-1} - \tanh[\theta_{i,t}(s_{l,t-1})]s_{l,t-1}\Big)P(s_{i,t}, s_{l,t-1}) \tag{202}$$

$$= \langle\tanh[\theta_{i,t}(s_{l,t-1})]s_{l,t-1}\rangle_P - \langle s_{i,t}s_{l,t-1}\rangle_P \tag{203}$$

$$= \langle s_{i,t}s_{l,t-1}\rangle_{(Q\cdot P)} - \langle s_{i,t}s_{l,t-1}\rangle_P = 0, \tag{204}$$

where $\langle s_{i,t}s_{l,t-1}\rangle_{(Q\cdot P)} = \sum_{s_{i,t},s_{l,t-1}} s_{i,t}s_{l,t-1}Q(s_{i,t}|s_{l,t-1})P(s_{l,t-1})$, and we used the equivalence $\tanh[\theta_{i,t}(s_{l,t-1})] = \sum_{s_{i,t}} s_{i,t}\frac{\mathrm{e}^{s_{i,t}\theta_{i,t}(s_{l,t-1})}}{2\cosh\theta_{i,t}(s_{l,t-1})}$.

This equation states that the closest factorized model has its first and second order moments equal to the first moments of the target distribution P. That is, $\langle s_{i,t}\rangle_Q = \langle s_{i,t}\rangle_P = m_{i,t}$ and $\langle s_{i,t}s_{l,t-1}\rangle_{(Q\cdot P)} = \langle s_{i,t}s_{l,t-1}\rangle_P = D_{il,t} + m_{i,t}m_{l,t-1}$. This is equivalent to having the marginalized distribution for spins i and l equal to the model Q, i.e., $P(s_{i,t}, s_{l,t-1}) = Q(s_{i,t}, s_{l,t-1})$. If we assume that the distribution P is close to Q, we can compute $P(s_{i,t}, s_{l,t-1})$ as an expansion with respect to α of the probability distribution:

$$P_\alpha(s_{i,t}, s_{l,t-1}) = \sum_{\substack{\mathbf{s}_{\backslash l,t-1}\\ \mathbf{s}_{t-2}}} P_\alpha(s_{i,t}|\mathbf{s}_{t-1})P_\alpha(s_{l,t-1}|\mathbf{s}_{t-2})P(\mathbf{s}_{\backslash l,t-1}|\mathbf{s}_{t-2})P(\mathbf{s}_{t-2}), \tag{205}$$

with

$$P_\alpha(s_{i,t}|\mathbf{s}_{t-1}) = \frac{\mathrm{e}^{s_{i,t}h_{i,t}(\alpha)}}{2\cosh h_{i,t}(\alpha)} \tag{206}$$

$$h_{i,t}(\alpha) = (1-\alpha)\theta_{i,t}(s_{l,t-1}) + \alpha(H_i + \sum_j J_{ij}s_{j,t-1}), \tag{207}$$

and

$$P_\alpha(s_{l,t-1}|\mathbf{s}_{t-2}) = \frac{\mathrm{e}^{s_{l,t-1}h_{l,t-1}(\alpha)}}{2\cosh h_{l,t-1}(\alpha)}, \tag{208}$$

$$h_{l,t-1}(\alpha) = (1-\alpha)\Theta_{l,t-1} + \alpha(H_l + \sum_n J_{ln}s_{n,t-2}). \tag{209}$$

When α is set to zero, $P_{\alpha=0}(s_{i,t}, s_{l,t-1}) = Q(s_{i,t}, s_{l,t-1})$, whereas when $\alpha = 1$, $P_{\alpha=1}(s_{i,t}, s_{l,t-1}) = P(s_{i,t}, s_{l,t-1})$. We approximate the values of $\Theta_{i,t}, \Theta_{l,t-1}, \Delta_{il,t}$ as follows:

$$\frac{\partial P_\alpha(s_{i,t}, s_{l,t-1})}{\partial\alpha} = \sum_{\substack{\mathbf{s}_{\backslash l,t-1}\\ \mathbf{s}_{t-1}}} \Big((s_{i,t} - \tanh h_{i,t}(\alpha))(-\theta_{i,t}(s_{l,t-1}) + H_i + \sum_j J_{ij}s_{j,t-1}) \tag{210}$$

$$+ (s_{l,t-1} - \tanh h_{l,t-1}(\alpha))(-\Theta_{l,t-1} + H_l + \sum_n J_{ln}s_{n,t-2})\Big) \tag{211}$$

$$\cdot P_\alpha(s_{i,t}|\mathbf{s}_{t-1})P_\alpha(s_{l,t-1}|\mathbf{s}_{t-2})P(\mathbf{s}_{\backslash l,t-1}|\mathbf{s}_{t-2})P(\mathbf{s}_{t-2}), \tag{212}$$

$$\frac{\partial P_\alpha(s_{i,t}, s_{l,t-1})}{\partial\alpha}\Big|_{\alpha=0} = \Big((s_{i,t} - \tanh\theta_{i,t}(s_{l,t-1}))(-\theta_{i,t}(s_{l,t-1}) + J_{il}(s_{l,t-1} - m_{l,t-1}) + H_i + \sum_j J_{ij}m_{j,t-1}) \tag{213}$$

$$+ (s_{l,t-1} - m_{l,t-1})(-\Theta_{l,t-1} + H_l + \sum_n J_{ln}m_{n,t-2})\Big)Q(s_{i,t}, s_{l,t-1}). \tag{214}$$

From here, we obtain the nMF equations:

$$\theta_{i,t}(s_{l,t-1}) \approx H_i + \sum_j J_{ij}m_{j,t-1} + J_{il}(s_{l,t-1} - m_{l,t-1}), \tag{215}$$

$$\Theta_{l,t-1} \approx H_l + \sum_n J_{ln}m_{n,t-1}. \tag{216}$$

The second order expressions are obtained by expanding:

$$\frac{\partial^2 P_\alpha(s_{i,t}, s_{l,t-1})}{\partial\alpha^2} = \sum_{\substack{\mathbf{s}_{\backslash l,t-1}\\ \mathbf{s}_{t-1}}} \Big(- (1 - \tanh^2 h_{i,t}(\alpha))(-\theta_{i,t}(s_{l,t-1}) + H_i + \sum_j J_{ij}s_{j,t-1})^2 \tag{217}$$

$$- (1 - \tanh^2 h_{l,t-1}(\alpha))(-\Theta_{l,t-1} + H_l + \sum_n J_{ln}s_{n,t-2})^2 \tag{218}$$

$$+ \big((s_{i,t} - \tanh h_{i,t}(\alpha))(-\theta_{i,t}(s_{l,t-1}) + H_i + \sum_j J_{ij}s_{j,t-1}) \tag{219}$$

$$+ (s_{l,t-1} - \tanh h_{l,t-1}(\alpha))(-\Theta_{l,t-1} + H_l + \sum_n J_{ln}s_{n,t-2})\big)^2\Big) \tag{220}$$

$$\cdot P_\alpha(s_{i,t}|\mathbf{s}_{t-1})P_\alpha(s_{l,t-1}|\mathbf{s}_{t-2})P(\mathbf{s}_{\backslash l,t-1}|\mathbf{s}_{t-2})P(\mathbf{s}_{t-2}), \tag{221}$$

$$\frac{\partial^2 P_\alpha(s_{i,t}, s_{l,t-1})}{\partial\alpha^2}\Big|_{\alpha=0} = \Big(- 2\tanh\theta_{i,t}(s_{l,t-1})(s_{i,t} - \tanh\theta_{i,t}(s_{l,t-1}))\big(W_{i,t}^2 + V_{ii,t}\big) \tag{222}$$

$$- 2m_{l,t-1}(s_{l,t-1} - m_{l,t-1})\big(W_{l,t-1}^2 + V_{ll,t-1}\big) \tag{223}$$

$$+ 2(s_{i,t} - \tanh\theta_{i,t}(s_{l,t-1}))(s_{l,t-1} - m_{l,t-1})\big(W_{i,t}W_{l,t-1} \tag{224}$$

$$+ \sum_{j\neq l,n} J_{ij}J_{ln}D_{jn,t-1}\big)\Big)Q(s_{i,t}, s_{l,t-1}), \tag{225}$$

where $V_{ii,t} = \sum_{j\neq l,n\neq l} J_{ij}J_{in}C_{jn,t-1}$, $V_{ll,t-1} = \sum_{mn} J_{lm}J_{ln}C_{mn,t-2}$, $W_{i,t} = -\theta_{i,t}(s_{l,t-1}) + H_i + \sum_j J_{ij}m_{j,t-1} + J_{il}(s_{l,t-1} - m_{l,t-1})$ and $W_{l,t-1} = -\Theta_{l,t-1} + H_l + \sum_n J_{ln}m_{n,t-1}$. Note that the first term in the second equation comes from the combination of the term in the first line with the squared term in the third line of the first equation. Similarly, the second term in the second equation derives from the combination of the second line and the squared term in the fourth line of the first equation.

We know that $\big[\alpha^2 W_{i,t}^2\big]_{\alpha=1} = 0 + \big[\mathcal{O}(\alpha^4)\big]_{\alpha=1}$, that $\big[\alpha^2 W_{l,t-1}^2\big]_{\alpha=1} = 0 + \big[\mathcal{O}(\alpha^4)\big]_{\alpha=1}$ and $\big[\alpha^2 W_{i,t}W_{l,t-1}\big]_{\alpha=1} = 0 + \big[\mathcal{O}(\alpha^4)\big]_{\alpha=1}$. This allows us to dismiss terms of the equations above. Then, grouping together the terms that

multiply $(s_{i,t} - \tanh\theta_{i,t}(s_{l,t-1}))$ and those that do not, we obtain the TAP equations:

$$\theta_{i,t}(s_{l,t-1}) = H_i + \sum_j J_{ij} m_{j,t-1} + \left(J_{il} + \sum_{j \neq l,n} J_{ij} J_{ln} D_{jn,t-1} \right) (s_{l,t-1} - m_{l,t-1}) \tag{226}$$

$$- \tanh\left[\theta_{i,t}(s_{l,t-1})\right] \sum_{jn \neq l} J_{ij} J_{in} C_{jn,t-1} + \left[\mathcal{O}(\alpha^2)\right]_{\alpha=1}, \tag{227}$$

$$\Theta_{l,t-1} = H_l + \sum_n J_{ln} m_{n,t-2} - m_{l,t-1} \sum_{mn} J_{lm} J_{ln} C_{mn,t-2} + \left[\mathcal{O}(\alpha^2)\right]_{\alpha=1}. \tag{228}$$

With these parameters we can compute the TAP approximations of the system's sufficient statistics as follows:

$$m_{i,t} \approx \sum_{s_{l,t-1}} \tanh[\theta_{i,t}(s_{l,t-1})] Q(s_{l,t-1}), \tag{229}$$

$$m_{l,t-1} \approx \tanh \Theta_{l,t-1}, \tag{230}$$

$$D_{il,t} \approx \sum_{s_{l,t-1}} (\tanh[\theta_{i,t}(s_{l,t-1})] - m_{i,t})(s_{l,t-1} - m_{l,t-1}) Q(s_{l,t-1}), \tag{231}$$

where $Q(s_{l,t-1}) = P(s_{l,t-1}) = \frac{1+s_{l,t-1}m_{l,t-1}}{2}$ is the factorised distribution for $s_{l,t-1}$.

Pairwise equal-time spin correlations

We can use a similar approximation in the case above to compute equal-time spin correlations. We do this by substituting $s_{l,t-1}$ in the approximation above by $s_{k,t}$. As $s_{i,t}$, $s_{k,t}$ are conditionally independent, we can assume that $s_{k,t}$ is computed first and that then $s_{i,t}$ is computed conditioned on $s_{k,t}$, (i.e., consider $P(s_{i,t}|s_{k,t}, \mathbf{s}_{t-1}) = P(s_{i,t}|\mathbf{s}_{t-1})$). In this approximation we consider a manifold $\mathcal{Q}$ with a pairwise probability distribution:

$$Q(s_{i,t}, s_{k,t}) = Q(s_{i,t}|s_{k,t}) Q(s_{k,t}) = \frac{\mathrm{e}^{s_{i,t}\theta_{i,t}(s_{k,t})}}{2\cosh\theta_{i,t}(s_{k,t})} \frac{\mathrm{e}^{s_{k,t}\Theta_{k,t}}}{2\cosh\Theta_{k,t}}, \tag{232}$$

where $\theta_{i,t}(s_{k,t}) = \Theta_{i,t} + \Delta_{ik,t} s_{k,t}$. Here $Q(s_{k,t})$ is the independent probability distribution for $s_{k,t}$ computed as in 3, and $Q(s_{i,t}|s_{k,t})$ is a conditional probability distribution we use to construct the pairwise probability distribution $Q(s_{i,t}, s_{k,t})$ using the chain rule.

We use this pairwise model to approximate the distribution

$$P(s_{i,t}, s_{k,t}) = \sum_{\mathbf{s}_{t-1}} P(s_{i,t}, s_{k,t}, \mathbf{s}_{t-1}) = \sum_{\mathbf{s}_{t-1}} P(s_{i,t}|\mathbf{s}_{t-1}) P(s_{k,t}, \mathbf{s}_{t-1}). \tag{233}$$

As in previous cases, we want to find an approximation of the probability distribution at time t that minimizes the relative entropy

$$D(P(s_{i,t}, s_{k,t}) || Q(s_{i,t}, s_{k,t})) = \sum_{\substack{s_{i,t} \\ s_{k,t}}} P(s_{i,t}, s_{k,t}) \log \frac{P(s_{i,t}, s_{k,t})}{Q(s_{i,t}, s_{k,t})}. \tag{234}$$

Specifically, the mean-field approximation that minimizes the relative entropy is the one that satisfies

$$\frac{\partial D(P(s_{i,t}, s_{k,t}) || Q(s_{i,t}, s_{k,t}))}{\partial \Theta_{i,t}} = - \sum_{\substack{s_{i,t} \\ s_{k,t}}} \Big(s_{i,t} - \tanh[\theta_{i,t}(s_{k,t})]) \Big) P(s_{i,t}, s_{k,t}) \tag{235}$$

$$= \langle s_{i,t} \rangle_Q - \langle s_{i,t} \rangle_P = 0, \tag{236}$$

$$\frac{\partial D(P(s_{i,t}, s_{k,t}) || Q(s_{i,t}, s_{k,t}))}{\partial \Delta_{ik,t}} = - \sum_{\substack{s_{i,t} \\ s_{k,t}}} \Big(s_{i,t} s_{k,t} - \tanh[\theta_{i,t}(s_{k,t})]) s_{k,t} \Big) P(s_{i,t}, s_{k,t}) \tag{237}$$

$$= \langle s_{i,t} s_{k,t} \rangle_{(Q \cdot P)} - \langle s_{i,t} s_{k,t} \rangle_P = 0, \tag{238}$$

where $\langle s_{i,t}s_{k,t}\rangle_{(Q\cdot P)} = \sum_{s_{i,t},s_{k,t}} Q(s_{i,t}|s_{k,t})P(s_{k,t})$.

This equation states that the closest factorized model has its first moments equal to the first and second order moments of the target distribution P. That is, $\langle s_{i,t}\rangle_Q = \langle s_{i,t}\rangle_P = m_{i,t}$ and $\langle s_{i,t}s_{k,t}\rangle_{(Q\cdot P)} = \langle s_{i,t}s_{k,t}\rangle_P = C_{ik,t} + m_{i,t}m_{k,t}$. This is equivalent to having the marginalized distribution for spins i and l equal to the model Q, i.e. $P(s_{i,t}, s_{k,t}) = Q(s_{i,t}, s_{k,t})$. If we assume that the distribution P is close to Q, we can compute $P(s_{i,t}, s_{k,t})$ as an expansion with respect to α of the probability distribution:

$$P_\alpha(s_{i,t}, s_{k,t}) = \sum_{\mathbf{s}_{t-1}} P_\alpha(s_{i,t}|s_{k,t}, \mathbf{s}_{t-1})P_\alpha(s_{k,t}|\mathbf{s}_{t-1})P(\mathbf{s}_{t-1}), \tag{239}$$

with

$$P_\alpha(s_{i,t}|s_{k,t}, \mathbf{s}_{t-1}) = \frac{\mathrm{e}^{s_{i,t}h_{i,t}(\alpha)}}{2\cosh h_{i,t}(\alpha)} \tag{240}$$

$$h_{i,t}(\alpha) = (1-\alpha)\theta_{i,t}(s_{k,t}) + \alpha(H_i + \sum_j J_{ij}s_{j,t-1}), \tag{241}$$

and

$$P_\alpha(s_{k,t}|\mathbf{s}_{t-1}) = \frac{\mathrm{e}^{s_{k,t}h_{k,t}(\alpha)}}{2\cosh h_{k,t}(\alpha)}, \tag{242}$$

$$h_{k,t}(\alpha) = (1-\alpha)\Theta_{k,t} + \alpha(H_k + \sum_l J_{kl}s_{l,t-1}). \tag{243}$$

When α is set to zero, $P_{\alpha=0}(s_{i,t}, s_{k,t}) = Q(s_{i,t}, s_{k,t})$, whereas when $\alpha = 1$, $P_{\alpha=1}(s_{i,t}, s_{k,t}) = P(s_{i,t}, s_{k,t})$. We approximate the values of $\Theta_{i,t}, \Theta_{k,t}, \Delta_{ik,t}$ as follows:

$$\frac{\partial P_\alpha(s_{i,t}, s_{k,t})}{\partial\alpha} = \sum_{\mathbf{s}_{t-1}} \Big((s_{i,t} - \tanh h_{i,t}(\alpha))(-\theta_{i,t}(s_{k,t}) + H_i + \sum_j J_{ij}s_{j,t-1}) \tag{244}$$

$$+ (s_{k,t} - \tanh h_{k,t}(\alpha))(-\Theta_{k,t} + H_k + \sum_l J_{kl}s_{l,t-1})\Big) \tag{245}$$

$$\cdot P_\alpha(s_{i,t}|s_{k,t}, \mathbf{s}_{t-1})P_\alpha(s_{k,t}|\mathbf{s}_{t-1})P(\mathbf{s}_{t-1}), \tag{246}$$

$$\frac{\partial P_\alpha(s_{i,t}, s_{k,t})}{\partial\alpha}\Big|_{\alpha=0} = \Big((s_{i,t} - \tanh\theta_{i,t}(s_{k,t}))(-\theta_{i,t}(s_{k,t}) + H_i + \sum_j J_{ij}m_{j,t-1}) \tag{247}$$

$$+ (s_{k,t} - m_{k,t})(-\Theta_{k,t} + H_k + \sum_l J_{kl}m_{l,t-1})\Big)Q(s_{i,t}, s_{k,t}). \tag{248}$$

From here, we obtain the nMF expansions:

$$\theta_{i,t}(s_{k,t}) = H_i + \sum_j J_{ij}m_{j,t-1} + \big[\mathcal{O}(\alpha^1)\big]_{\alpha=1}, \tag{249}$$

$$\Theta_{k,t} = H_k + \sum_l J_{kl}m_{l,t-1} + \big[\mathcal{O}(\alpha^1)\big]_{\alpha=1}. \tag{250}$$

The second order expressions are obtained by expanding:

$$\frac{\partial^2 P_\alpha(s_{i,t}, s_{k,t})}{\partial\alpha^2} = \sum_{\mathbf{s}_{t-1}} \Big(-(1-\tanh^2 h_{i,t}(\alpha))(-\theta_{i,t}(s_{k,t}) + H_i + \sum_j J_{ij}s_{j,t-1})^2 \tag{251}$$

$$-(1-\tanh^2 h_{k,t}(\alpha))(-\Theta_{k,t} + H_k + \sum_l J_{kl}s_{l,t-1})^2 \tag{252}$$

$$+ \big((s_{i,t} - \tanh h_{i,t}(\alpha))(-\theta_{i,t}(s_{k,t}) + H_i + \sum_j J_{ij}s_{j,t-1}) \tag{253}$$

$$+ (s_{k,t} - \tanh h_{k,t}(\alpha))(-\Theta_{k,t} + H_k + \sum_l J_{kl}s_{n,t-1})\big)^2\Big) \tag{254}$$

$$\cdot P_\alpha(s_{i,t}|s_{k,t}, \mathbf{s}_{t-1})P_\alpha(s_{k,t}|\mathbf{s}_{t-1})P(\mathbf{s}_{t-1}), \tag{255}$$

$$\frac{\partial^2 P_\alpha(s_{i,t}, s_{k,t})}{\partial \alpha^2}\Big|_{\alpha=0} = \Big(-2\tanh\theta_{i,t}(s_{k,t})(s_{i,t} - \tanh\theta_{i,t}(s_{k,t}))\big(W_{i,t}^2 + V_{ii,t}\big) \tag{256}$$

$$- 2m_{k,t}(s_{k,t} - m_{k,t})\big(W_{k,t}^2 + V_{kk,t}\big) \tag{257}$$

$$+ 2(s_{i,t} - \tanh\theta_{i,t}(s_{k,t}))(s_{k,t} - m_{k,t})\big(W_{i,t}W_{k,t} + V_{ik,t}\big)\Big) Q(s_{i,t}, s_{k,t}), \tag{258}$$

where $V_{ik,t} = \sum_{jl} J_{ij} J_{kl} C_{jl,t-1}$ and $W_{i,t} = -\theta_{i,t} + H_i + \sum_j J_{ij} m_{j,t-1}$. Again, note that the first term in the second equation comes from the combination of the term in the first line with the squared term in the third line of the first equation. Similarly, the second term in the second equation derives from the combination of the second line and the squared term in the fourth line of the first equation.

We know that $\left[\alpha^2 W_{i,t}^2\right]_{\alpha=1} = 0 + \left[\mathcal{O}(\alpha^4)\right]_{\alpha=1}$, that $\left[\alpha^2 W_{k,t}^2\right]_{\alpha=1} = 0 + \left[\mathcal{O}(\alpha^4)\right]_{\alpha=1}$ and $\left[\alpha^2 W_{i,t} W_{k,t}\right]_{\alpha=1} = 0 + \left[\mathcal{O}(\alpha^4)\right]_{\alpha=1}$. This allows us to dismiss terms of the equations above. Again, grouping terms that contain $(s_{i,t} - \tanh\theta_{i,t}(s_{l,t-1}))$ and those that not we obtain the follwoing Plefka expansions for the TAP approximations:

$$\theta_{i,t}(s_{k,t}) = H_i + \sum_j J_{ij} m_{j,t-1} + (s_{k,t} - m_{k,t}) \sum_{jl} J_{ij} J_{kl} C_{jl,t-1} \tag{259}$$

$$- \tanh\left[\theta_{i,t}(s_{k,t})\right] \sum_{jl} J_{ij} J_{il} C_{jl,t-1} + \left[\mathcal{O}(\alpha^2)\right]_{\alpha=1}, \tag{260}$$

$$\Theta_{k,t} = H_k + \sum_l J_{kl} m_{l,t-1} - m_{k,t} \sum_{jl} J_{kj} J_{kl} C_{jl,t-1} + \left[\mathcal{O}(\alpha^2)\right]_{\alpha=1}. \tag{261}$$

With these parameters we can the TAP approximations of the system's statistics as follows:

$$m_{i,t} \approx \sum_{s_{k,t}} \tanh[\theta_{i,t}(s_{k,t})] Q(s_{k,t}), \tag{262}$$

$$m_{k,t} \approx \tanh\Theta_{k,t}, \tag{263}$$

$$C_{ik,t} \approx \sum_{s_{k,t}} (\tanh[\theta_{i,t}(s_{k,t})] - m_{i,t})(s_{k,t} - m_{k,t}) Q(s_{k,t}), \tag{264}$$

where $Q(s_{k,t}) = P(s_{k,t}) = \frac{1+s_{k,t}m_{k,t}}{2}$ is the factorised probability of unit $s_{k,t}$.

Supplementary Note 6: Solution of the asymmetric kinetic Sherrington-Kirkpatrick model

The infinite kinetic Ising model with Gaussian couplings used in this article is generally referred in its symmetric version as the Sherrington-Kirkpatrick (SK) model. The SK model behaviour is well studied in statistical mechanics, and its solution can be obtained using the replica trick [5]. In the symmetric case, dynamics can be represented as a bipartite network that can also be solved using the replica trick [6]. Here, we extend the solution to the kinetic, asymmetric version of the model. As this model does not have an equilibrium distribution or a free energy defined in classical terms as the SK model, we need to recur to an dynamical equivalent in the form of a generating functional.

Generating functional

We start with a kinetic Ising model

$$P(\mathbf{s}_t \mid \mathbf{s}_{t-1}) = \prod_i \frac{\mathrm{e}^{\beta s_{i,t} h_{i,t}}}{2\cosh(\beta h_{i,t})}, \tag{265}$$

$$h_{i,t} = H_{i,t} + \sum_j J_{ij} s_{j,t-1}, \tag{266}$$

where β is the inverse temperature, $H_{i,t}$ are time-varying fields and J_{ij} couplings. The results apply similarly to fixed fields.

The probability of a specific trajectory $\mathbf{s}_{0:t}$, is defined as

$$P(\mathbf{s}_{0:t}) = \prod_{u=1}^{t} P(\mathbf{s}_u|\mathbf{s}_{u-1}) P(\mathbf{s}_0) \tag{267}$$

$$= \exp\left(\sum_u \left(\sum_i s_{i,u}\left(\beta H_{i,u} + g_{i,u} + \beta \sum_j J_{ij} s_{j,u-1}\right)\right)\right. \tag{268}$$

$$\left. - \sum_{i,u} \log 2\cosh\left(\beta\left(H_{i,u} + \sum_j J_{ij} s_{j,u-1}\right)\right)\right) P(\mathbf{s}_0). \tag{269}$$

Instead of a partition function, the distribution of the trajectories of the asymmetric SK model can be described by defining a generating functional or a dynamical partition function:

$$Z_t(\mathbf{g}) = \sum_{\mathbf{s}_{0:t}} \exp\left(\sum_{iu} g_{i,u} s_{i,u}\right) P(\mathbf{s}_{0:t}) \tag{270}$$

$$= \sum_{\mathbf{s}_{0:t}} \exp\left(\sum_u \left(\sum_i s_{i,u}\left(\beta H_{i,u} + g_{i,u} + \beta \sum_j J_{ij} s_{j,u-1}\right)\right)\right. \tag{271}$$

$$\left. - \sum_{i,u} \log 2\cosh\left(\beta\left(H_{i,u} + \sum_j J_{ij} s_{j,u-1}\right)\right)\right) P(\mathbf{s}_0). \tag{272}$$

Note that $Z_t(\mathbf{0}) = 1$. For simplicity we will assume an initial distribution with a single possible state $\mathbf{s}_0$, i.e. $P(\mathbf{s}'_0) = \delta_{\mathbf{s}'_0,\mathbf{s}_0}$, which allows us to drop the $P(\mathbf{s}_0)$ term in the equation above. In the $t \to \infty$ limit, the logarithmic dynamical partition function converges to the large deviation function

$$\lim_{t\to\infty} \frac{1}{t} \log Z_t(\mathbf{g}) = \varphi(\mathbf{g}), \tag{273}$$

which plays the role of a free-energy function for trajectories [7].

The generating functional can be used to compute moments of the system as:

$$m_{i,u} = \lim_{\mathbf{g}\to\mathbf{0}} \frac{\partial Z_t(\mathbf{g})}{\partial g_{i,u}} = \lim_{\mathbf{g}\to\mathbf{0}} \langle s_{i,u}\rangle_{\mathbf{g}} = \langle s_{i,u}\rangle, \tag{274}$$

$$R_{ij,uv} = \lim_{\mathbf{g}\to\mathbf{0}} \frac{\partial^2 Z_t(\mathbf{g})}{\partial g_{i,u}\partial g_{j,v}} = \lim_{\mathbf{g}\to\mathbf{0}} \langle s_{i,u} s_{j,v}\rangle_{\mathbf{g}} = \langle s_{i,u} s_{j,v}\rangle, \tag{275}$$

where the brackets are defined as

$$\langle f(\mathbf{s})\rangle_{\mathbf{g}} =\sum_{\mathbf{s}_{0:t}} f(\mathbf{s}) \exp\left(\sum_{iu} g_{i,u}s_{i,u}\right)P(\mathbf{s}_{0:t}), \tag{276}$$

$$\langle f(\mathbf{s})\rangle =\sum_{\mathbf{s}_{0:t}} f(\mathbf{s})P(\mathbf{s}_{0:t}). \tag{277}$$

It also allows us to derive identities that will be helpful in eliminating spurious solutions

$$\lim_{\mathbf{g}\to\mathbf{0}} \frac{\partial Z_t(\mathbf{g})}{\partial H_{i,u}} = \beta\left(m_{i,u} - m_{i,u}\right) =0, \tag{278}$$

$$\lim_{\mathbf{g}\to\mathbf{0}} \frac{\partial^2 Z_t(\mathbf{g})}{\partial H_{i,u}\partial H_{j,v}} = \beta\left(\frac{\partial m_{i,u}}{\partial H_{j,v}} - \frac{\partial m_{i,u}}{\partial H_{j,v}}\right) =0. \tag{279}$$

Path integral

In the asymmetric SK model, the couplings J_{ij} are quenched variables with a Gaussian distribution function

$$P(J_{ij}) = \frac{1}{\sqrt{2\pi J_\sigma^2/N}} \exp\left(-\frac{1}{2J_\sigma^2/N}\left(J_{ij} - \frac{J_0}{N}\right)^2\right), \tag{280}$$

where the mean and the variance are proportional to $1/N$.

In the thermodynamic limit, one can study the system by computing the configurational average

$$[Z_t(\mathbf{g})] = \int \prod_{ij} \mathrm{d}J_{ij} P(J_{ij}) Z_T(\mathbf{g}). \tag{281}$$

The quenched average can be solved using path integral methods, by inserting an appropriate delta integral for the effective fields of each unit

$$1 = \int \mathrm{d}\boldsymbol{\theta} \prod_{i,u} \delta(\theta_{i,u} - \beta H_{i,u} - \beta \sum_j J_{ij}s_{j,u-1}) = \frac{1}{(2\pi)^{Nt}} \int \mathrm{d}\boldsymbol{\theta}\mathrm{d}\hat{\boldsymbol{\theta}} \exp\left(\sum_{iu} \mathrm{i}\hat{\theta}_{i,t}(\theta_{i,t} - \beta H_{i,u} - \beta \sum_j J_{ij}s_{j.t-1})\right). \tag{282}$$

where $\boldsymbol{\theta}$ represent the effective fields of units and $\hat{\boldsymbol{\theta}}$ are conjugates of these fields.

By inserting the above equation, the configurational average is written as

$$[Z_t(\mathbf{g})] =\frac{1}{(2\pi)^{Nt}} \int \mathrm{d}\boldsymbol{\theta}\mathrm{d}\hat{\boldsymbol{\theta}} \prod_{ij} \mathrm{d}J_{ij} P(J_{ij}) \sum_{\mathbf{s}_{1:t}} \exp\Bigg(\sum_{iu} s_{i,u}(g_{i,u} + \theta_{i,u}) - \sum_{iu} \log 2\cosh\left(\theta_{i,u}\right) \tag{283}$$

$$+ \sum_{iu} \mathrm{i}\hat{\theta}_{i,u}(\theta_{i,u} - \beta H_{i,u} - \beta \sum_j J_{ij}s_{j,u-1})\Bigg) \tag{284}$$

$$=\frac{1}{(2\pi)^{Nt}} \int \mathrm{d}\boldsymbol{\theta}\mathrm{d}\hat{\boldsymbol{\theta}} \sum_{\mathbf{s}_{1:t}} \exp\Bigg(\sum_{iu} s_{i,u}(g_{i,u} + \theta_{i,u}) - \sum_{iu} \log 2\cosh\left(\theta_{i,u}\right) + \sum_{iu} \mathrm{i}\hat{\theta}_{i,u}(\theta_{i,u} - \beta H_{i,u}) \tag{285}$$

$$- \sum_{iu} \mathrm{i}\frac{\beta J_0}{N}\hat{\theta}_{i,u} \sum_j s_{j,u-1} + \sum_{ij} \frac{\beta^2 J_\sigma^2}{2N}\left(\mathrm{i}\sum_u \hat{\theta}_{i,u}s_{j,u-1}\right)^2\Bigg) \tag{286}$$

$$=\frac{1}{(2\pi)^{Nt}} \int \mathrm{d}\boldsymbol{\theta}\mathrm{d}\hat{\boldsymbol{\theta}} \sum_{\mathbf{s}_{1:t}} \exp\Bigg(\sum_{iu} s_{i,u}(g_{i,u} + \theta_{i,u}) - \sum_{iu} \log 2\cosh\left(\theta_{i,u}\right) + \sum_{iu} \mathrm{i}\hat{\theta}_{i,u}(\theta_{i,u} - \beta H_{i,u}) \tag{287}$$

$$- N\beta J_0 \sum_u \frac{1}{N}\sum_i \mathrm{i}\hat{\theta}_{i,u} \frac{1}{N}\sum_j s_{j,u-1} + N\frac{\beta^2 J_\sigma^2}{2}\sum_{uv} \frac{1}{N}\sum_i \mathrm{i}\hat{\theta}_{i,u}\mathrm{i}\hat{\theta}_{i,v} \frac{1}{N}\sum_j s_{j,u-1}s_{j,v-1}\Bigg). \tag{288}$$

Gaussian integral

We want to simplify the expression above by introducing new variables that will become order parameters. We do so by introducing a double Gaussian integral with the form:

$$\exp\left(Cxy\right) = \exp\left(\frac{C}{2}\left(\frac{1}{2}(x+y)^2 + \frac{1}{2}(\mathrm{i}(x-y))^2\right)\right) \tag{289}$$

$$= \frac{C}{\pi}\int \mathrm{d}z_R \mathrm{d}z_I \exp\left(\frac{C}{2}\left(-\frac{1}{2}z_R^2 - \frac{1}{2}z_I^2 + (x+y)z_R + \mathrm{i}(x-y)z_I\right)\right) \tag{290}$$

$$= \frac{C}{\pi}\int \mathrm{d}z_R \mathrm{d}z_I \exp\left(\frac{C}{2}\left(-\frac{1}{2}z_R^2 - \frac{1}{2}z_I^2 + x(z_R + \mathrm{i}z_I) + y(z_R - \mathrm{i}z_I)\right)\right) \tag{291}$$

$$= \frac{C}{\pi}\int \mathrm{d}z_1 \mathrm{d}z_2 \exp\left(C\left(-z_1 z_2 + x z_1 + y z_2\right)\right), \tag{292}$$

where in the last step we applied a change of variables $z_1 = \frac{1}{2}(z_R + \mathrm{i}z_I), z_2 = \frac{1}{2}(z_R - \mathrm{i}z_I)$.

We apply the Gaussian integral above:

$$\exp\left(N\beta J_0 \frac{1}{N}\sum_j s_{j,u-1}\frac{1}{N}\sum_i(-\mathrm{i}\hat{\theta}_{i,u})\right) = \frac{N\beta J_0}{\pi}\int \mathrm{d}\mu_u \mathrm{d}m_{u-1}\exp\Bigg(-N\beta J_0 \mu_u m_{u-1} \tag{293}$$

$$+ \beta J_0 \mu_u \sum_j s_{j,u-1} - \beta J_0 m_{u-1}\sum_i \mathrm{i}\hat{\theta}_{i,u}\Bigg), \tag{294}$$

by using $z_1 = \mu_u$, $z_2 = m_{u-1}$, $x = \frac{1}{N}\sum_j s_{j,u-1}$, $y = \frac{1}{N}\sum_i(-\mathrm{i}\hat{\theta}_{i,u})$, and $C = N\beta J_0$. Similarly, we have

(295)

$$\exp\left(N\beta^2 J_\sigma^2 \frac{1}{N}\sum_j s_{j,u-1}s_{j,v-1}\frac{1}{N}\sum_i \mathrm{i}\hat{\theta}_{i,u}\mathrm{i}\hat{\theta}_{i,v}\right) = \frac{N\beta^2 J_\sigma^2}{\pi}\int \mathrm{d}\rho_{u,v}\mathrm{d}q_{u-1,v-1}\exp\Bigg(-N\beta^2 J_\sigma^2 \rho_{u,v} q_{u-1,v-1} \tag{296}$$

$$+ \beta^2 J_\sigma^2 \mu_u \sum_j s_{j,u-1} + \beta^2 J_\sigma^2 q_{u-1,v-1}\sum_i \mathrm{i}\hat{\theta}_{i,u}\Bigg). \tag{297}$$

With these transformations, we can rewrite

$$[Z_t(\mathbf{g})] = \frac{(N\beta J_0)^t (N\beta^2 J_\sigma^2)^t}{\pi^{2t}}\int \mathrm{d}\mathbf{m}\mathrm{d}\boldsymbol{\mu}\mathrm{d}\mathbf{q}\mathrm{d}\boldsymbol{\rho}\exp\Bigg(-N\beta J_0 \sum_u \mu_u m_{u-1} - N\beta^2 J_\sigma^2 \sum_{u>v}\rho_{u,v}q_{u-1,v-1} \tag{298}$$

$$+ \log\sum_{\mathbf{s}_{1:t}}\int \mathrm{d}\boldsymbol{\theta}\mathrm{d}\hat{\boldsymbol{\theta}}\mathrm{e}^{\Phi(\mathbf{s},\boldsymbol{\theta})+\Omega(\hat{\boldsymbol{\theta}},\boldsymbol{\theta})}\Bigg), \tag{299}$$

where the rest of the terms from the Gaussian integral are contained in the terms

$$\Phi(\mathbf{s},\boldsymbol{\theta}) = \sum_{iu}\left(g_{i,u} + \theta_{i,u}\right)s_{i,u} + \sum_{iu}\beta J_0 \mu_u s_{i,u-1} + \sum_{i,u>v}\beta^2 J_\sigma^2 \rho_{u,v}s_{i,u-1}s_{i,v-1} - \sum_{iu}\log 2\cosh\left(\theta_{i,u}\right), \tag{300}$$

$$\Omega(\hat{\boldsymbol{\theta}},\boldsymbol{\theta}) = \sum_{iu}(\theta_{i,u} - \beta H_{i,u} - \beta J_0 m_{u-1})\mathrm{i}\hat{\theta}_{i,u} + \frac{\beta^2 J_\sigma^2}{2}\sum_{i,u}\left(\mathrm{i}\hat{\theta}_{i,u}\right)^2 + \beta^2 J_\sigma^2 \sum_{i,u>v} q_{u-1,v-1}\mathrm{i}\hat{\theta}_{i,u}\mathrm{i}\hat{\theta}_{i,v} - Nt\log 2\pi. \tag{301}$$

We will see now that the values of $\mathbf{m}, \boldsymbol{\mu}, \mathbf{q}, \boldsymbol{\rho}$ will act as order parameters of the system.

Order parameters

The exponent of the above integrand is proportional to N, being it possible to evaluate the integral by steepest descent, giving the saddle-point solution as

$$[Z_t(\mathbf{g})] = \exp\left\{-N\beta J_0 \sum_u \mu_u m_{u-1} - N\beta^2 J_\sigma^2 \sum_{u>v}\rho_{u,v}q_{u-1,v-1} + \log\sum_{\mathbf{s}_{1:t}}\int \mathrm{d}\boldsymbol{\theta}\mathrm{d}\hat{\boldsymbol{\theta}}\mathrm{e}^{\Phi(\mathbf{s},\boldsymbol{\theta})+\Omega(\hat{\boldsymbol{\theta}},\boldsymbol{\theta})}\right\}, \tag{302}$$

where the values of $\mathbf{m}, \boldsymbol{\mu}, \mathbf{q}, \boldsymbol{\rho}$ are chosen to extremize (maximize or minimize) the quantity between the braces $\{\}$. Notice that integration over disordered connections has removed coupling between units and replaced it with same-unit temporal couplings ρ and varying effective fields, which are also independent between units, resulting in a mean-field solution where the activity of different spins is independent.

From here, knowing that $\lim_{\mathbf{g}\to\mathbf{0}} \frac{\partial [Z_t(\mathbf{g})]}{\partial g_{i,u}} = [\langle s_{i,u}\rangle]$, $\lim_{\mathbf{g}\to\mathbf{0}} \frac{\partial^2 [Z_t(\mathbf{g})]}{\partial g_{i,u}\partial g_{j,v}} = [\langle s_{i,u} s_{j,v}\rangle]$, and taking into account that $\lim_{\mathbf{g}\to\mathbf{0}} [Z_t(\mathbf{g})] = 1$ we can compute the order parameters of the system as

$$\lim_{\mathbf{g}\to\mathbf{0}} \frac{\partial\,[Z_t(\mathbf{g})]}{\partial g_{i,u}} = \lim_{\mathbf{g}\to\mathbf{0}} \frac{\sum_{\mathbf{s}_{1:t}} \int \mathrm{d}\boldsymbol{\theta}\mathrm{d}\hat{\boldsymbol{\theta}} s_{i,u} \mathrm{e}^{\Phi(\mathbf{s},\boldsymbol{\theta})+\Omega(\hat{\boldsymbol{\theta}},\boldsymbol{\theta})}}{\sum_{\mathbf{s}_{1:t}} \int \mathrm{d}\boldsymbol{\theta}\mathrm{d}\hat{\boldsymbol{\theta}} \mathrm{e}^{\Phi(\mathbf{s},\boldsymbol{\theta})+\Omega(\hat{\boldsymbol{\theta}},\boldsymbol{\theta})}} [Z_t(\mathbf{g})] = \langle s_{i,u}\rangle_* = [\langle s_{i,u}\rangle]\,, \tag{303}$$

$$\lim_{\mathbf{g}\to\mathbf{0}} \frac{\partial^2\,[Z_t(\mathbf{g})]}{\partial g_{i,u}\partial g_{j,v}} = \lim_{\mathbf{g}\to\mathbf{0}} \frac{\sum_{\mathbf{s}_{1:t}} \int \mathrm{d}\boldsymbol{\theta}\mathrm{d}\hat{\boldsymbol{\theta}} s_{i,u}s_{j,v} \mathrm{e}^{\Phi(\mathbf{s},\boldsymbol{\theta})+\Omega(\hat{\boldsymbol{\theta}},\boldsymbol{\theta})}}{\sum_{\mathbf{s}_{1:t}} \int \mathrm{d}\boldsymbol{\theta}\mathrm{d}\hat{\boldsymbol{\theta}} \mathrm{e}^{\Phi(\mathbf{s},\boldsymbol{\theta})+\Omega(\hat{\boldsymbol{\theta}},\boldsymbol{\theta})}} [Z_t(\mathbf{g})] = \langle s_{i,u}s_{j,v}\rangle_* = [\langle s_{i,u}s_{j,v}\rangle]\,, \tag{304}$$

$$\lim_{\mathbf{g}\to\mathbf{0}} \frac{\partial\,[Z_t(\mathbf{g})]}{\partial H_{i,u}} = \lim_{\mathbf{g}\to\mathbf{0}} \frac{\sum_{\mathbf{s}_{1:t}} \int \mathrm{d}\boldsymbol{\theta}\mathrm{d}\hat{\boldsymbol{\theta}} - \beta \mathrm{i}\hat{\theta}_{i,u} \mathrm{e}^{\Phi(\mathbf{s},\boldsymbol{\theta})+\Omega(\hat{\boldsymbol{\theta}},\boldsymbol{\theta})}}{\sum_{\mathbf{s}_{1:t}} \int \mathrm{d}\boldsymbol{\theta}\mathrm{d}\hat{\boldsymbol{\theta}} \mathrm{e}^{\Phi(\mathbf{s},\boldsymbol{\theta})+\Omega(\hat{\boldsymbol{\theta}},\boldsymbol{\theta})}} [Z_t(\mathbf{g})] = -\beta \left\langle \mathrm{i}\hat{\theta}_{i,u}\right\rangle_* = 0, \tag{305}$$

$$\lim_{\mathbf{g}\to\mathbf{0}} \frac{\partial^2\,[Z_t(\mathbf{g})]}{\partial H_{i,u}\partial H_{j,v}} = \lim_{\mathbf{g}\to\mathbf{0}} \frac{\sum_{\mathbf{s}_{1:t}} \int \mathrm{d}\boldsymbol{\theta}\mathrm{d}\hat{\boldsymbol{\theta}} \beta^2 \mathrm{i}\hat{\theta}_{i,u}\mathrm{i}\hat{\theta}_{j,v} \mathrm{e}^{\Phi(\mathbf{s},\boldsymbol{\theta})+\Omega(\hat{\boldsymbol{\theta}},\boldsymbol{\theta})}}{\sum_{\mathbf{s}_{1:t}} \int \mathrm{d}\boldsymbol{\theta}\mathrm{d}\hat{\boldsymbol{\theta}} \mathrm{e}^{\Phi(\mathbf{s},\boldsymbol{\theta})+\Omega(\hat{\boldsymbol{\theta}},\boldsymbol{\theta})}} [Z_t(\mathbf{g})] = \beta^2 \left\langle \mathrm{i}\hat{\theta}_{i,u}\mathrm{i}\hat{\theta}_{j,v}\right\rangle_* = 0, \tag{306}$$

where

$$\left\langle f(\mathbf{s},\hat{\boldsymbol{\theta}})\right\rangle_* = \frac{\sum_{\mathbf{s}_{1:t}} \int \mathrm{d}\boldsymbol{\theta}\mathrm{d}\hat{\boldsymbol{\theta}} f(\mathbf{s},\hat{\boldsymbol{\theta}}) \mathrm{e}^{\Phi(\mathbf{s},\boldsymbol{\theta})+\Omega(\hat{\boldsymbol{\theta}},\boldsymbol{\theta})}}{\sum_{\mathbf{s}_{1:t}} \int \mathrm{d}\boldsymbol{\theta}\mathrm{d}\hat{\boldsymbol{\theta}} \mathrm{e}^{\Phi(\mathbf{s},\boldsymbol{\theta})+\Omega(\hat{\boldsymbol{\theta}},\boldsymbol{\theta})}}. \tag{307}$$

Here we should note that, as there is no coupling between units, for $i \neq j$ we have a factorised solution $[\langle s_{i,u}s_{j,v}\rangle] = \langle s_{i,u}s_{j,v}\rangle_* = \langle s_{i,u}\rangle \langle s_{j,v}\rangle_*$.

To obtain the values of the order parameters, we extremize the contents of the brackets, finding

$$\lim_{\mathbf{g}\to\mathbf{0}} \frac{\partial \log [Z_t(\mathbf{g})]}{\partial \mu_{u+1}} = -N\beta J_0 m_u + \lim_{\mathbf{g}\to\mathbf{0}} \frac{\sum_{\mathbf{s}_{1:t}} \int \mathrm{d}\boldsymbol{\theta}\mathrm{d}\hat{\boldsymbol{\theta}} \beta J_0 s_{i,u} \mathrm{e}^{\Phi(\mathbf{s},\boldsymbol{\theta})+\Omega(\hat{\boldsymbol{\theta}},\boldsymbol{\theta})}}{\sum_{\mathbf{s}_{1:t}} \int \mathrm{d}\boldsymbol{\theta}\mathrm{d}\hat{\boldsymbol{\theta}} \mathrm{e}^{\Phi(\mathbf{s},\boldsymbol{\theta})+\Omega(\hat{\boldsymbol{\theta}},\boldsymbol{\theta})}} \tag{308}$$

$$=\beta J_0 \left(\sum_i \langle s_{i,u}\rangle_* - N m_u\right) = 0; \qquad m_u = \frac{1}{N}\sum_i [\langle s_{i,u}\rangle]\,, \tag{309}$$

$$\lim_{\mathbf{g}\to\mathbf{0}} \frac{\partial \log [Z_t(\mathbf{g})]}{\partial m_{u-1}} = -N\beta J_0 \mu_u + \lim_{\mathbf{g}\to\mathbf{0}} \frac{\sum_{\mathbf{s}_{1:t}} \int \mathrm{d}\boldsymbol{\theta}\mathrm{d}\hat{\boldsymbol{\theta}} - \beta J_0 \mathrm{i}\hat{\theta}_{i,u} \mathrm{e}^{\Phi(\mathbf{s},\boldsymbol{\theta})+\Omega(\hat{\boldsymbol{\theta}},\boldsymbol{\theta})}}{\sum_{\mathbf{s}_{1:t}} \int \mathrm{d}\boldsymbol{\theta}\mathrm{d}\hat{\boldsymbol{\theta}} \mathrm{e}^{\Phi(\mathbf{s},\boldsymbol{\theta})+\Omega(\hat{\boldsymbol{\theta}},\boldsymbol{\theta})}} \tag{310}$$

$$=-\beta J_0 \left(\sum_i \left\langle \mathrm{i}\hat{\theta}_{i,u}\right\rangle_* + N\mu_u\right) = 0; \qquad \mu_u = 0, \tag{311}$$

$$\lim_{\mathbf{g}\to\mathbf{0}} \frac{\partial \log [Z_t(\mathbf{g})]}{\partial \rho_{u+1,v+1}} = -N\beta^2 J_\sigma^2 q_{u,v} + \lim_{\mathbf{g}\to\mathbf{0}} \frac{\sum_{\mathbf{s}_{1:t}} \int \mathrm{d}\boldsymbol{\theta}\mathrm{d}\hat{\boldsymbol{\theta}} \beta^2 J_\sigma^2 s_{i,u}s_{i,v} \mathrm{e}^{\Phi(\mathbf{s},\boldsymbol{\theta})+\Omega(\hat{\boldsymbol{\theta}},\boldsymbol{\theta})}}{\sum_{\mathbf{s}_{1:t}} \int \mathrm{d}\boldsymbol{\theta}\mathrm{d}\hat{\boldsymbol{\theta}} \mathrm{e}^{\Phi(\mathbf{s},\boldsymbol{\theta})+\Omega(\hat{\boldsymbol{\theta}},\boldsymbol{\theta})}} \tag{312}$$

$$=N\beta^2 J_\sigma^2 \left(\sum_i \langle s_{i,u}s_{i,v}\rangle_* - N q_{uv}\right) = 0; \qquad q_{uv} = \frac{1}{N}\sum_i [\langle s_{i,u}s_{i,v}\rangle]\,, \tag{313}$$

$$\lim_{\mathbf{g}\to\mathbf{0}} \frac{\partial \log [Z_t(\mathbf{g})]}{\partial q_{u-1,v-1}} = -N\beta^2 J_\sigma^2 \rho_{u,v} + \lim_{\mathbf{g}\to\mathbf{0}} \frac{\sum_{\mathbf{s}_{1:t}} \int \mathrm{d}\boldsymbol{\theta}\mathrm{d}\hat{\boldsymbol{\theta}} \beta^2 J_\sigma^2 \mathrm{i}\hat{\theta}_{i,u}\mathrm{i}\hat{\theta}_{j,v} \mathrm{e}^{\Phi(\mathbf{s},\boldsymbol{\theta})+\Omega(\hat{\boldsymbol{\theta}},\boldsymbol{\theta})}}{\sum_{\mathbf{s}_{1:t}} \int \mathrm{d}\boldsymbol{\theta}\mathrm{d}\hat{\boldsymbol{\theta}} \mathrm{e}^{\Phi(\mathbf{s},\boldsymbol{\theta})+\Omega(\hat{\boldsymbol{\theta}},\boldsymbol{\theta})}} \tag{314}$$

$$=N\beta^2 J_\sigma^2 \left(\sum_i \langle \mathrm{i}\theta_{i,u}\mathrm{i}\theta_{i,v}\rangle_* - N\rho_{uv}\right) = 0; \qquad \rho_{uv} = 0. \tag{315}$$

Saddle-point solution

After solving the saddle-point integral, we have the following dynamical partition function

$$[Z_t(\mathbf{g})] = \sum_{\mathbf{s}_{1:t}} \int \mathrm{d}\boldsymbol{\theta}\mathrm{d}\hat{\boldsymbol{\theta}} \mathrm{e}^{\Phi(\mathbf{s},\boldsymbol{\theta})+\Omega(\hat{\boldsymbol{\theta}},\boldsymbol{\theta})}. \tag{316}$$

At this point, we want to remove the effective conjugate fields $\hat{\boldsymbol{\theta}}$ by recovering a delta function. We first rewrite

$$\mathrm{e}^{\Omega(\hat{\boldsymbol{\theta}},\boldsymbol{\theta})} = \frac{1}{(2\pi)^{Nt}} \exp\left(\sum_{iu} (\theta_{i,u} - \beta H_{i,u} - \beta J_0 m_{u-1}) \mathrm{i}\hat{\theta}_{i,u} + \frac{\beta^2 J_\sigma^2}{2} \sum_{i,uv} q_{u-1,v-1} \mathrm{i}\hat{\theta}_{i,u} \mathrm{i}\hat{\theta}_{i,v} \right), \tag{317}$$

where we defined $q_{u-1,u-1} = 1$ and $q_{u-1,v-1} = q_{v-1,u-1}$. We can remove the quadratic terms of $\hat{\boldsymbol{\theta}}$ by applying a multivariate Gaussian integral of the form

$$\mathrm{e}^{\frac{1}{2}\sum_{uv} K_{uv} x_u x_v} = \frac{1}{\sqrt{(2\pi)^t |K^{-1}|}} \int \mathrm{d}\mathbf{z} \mathrm{e}^{-\frac{1}{2}\sum_{uv} K_{uv} z_u z_v + \sum_{uv} K_{uv} x_u z_v}. \tag{318}$$

For $x_u = -\beta J_\sigma \mathrm{i}\hat{\theta}_{i,u}$ and $K_{uv} = q_{u-1,v-1}$, we get

$$\int \mathrm{d}\hat{\boldsymbol{\theta}} \mathrm{e}^{\Omega(\hat{\boldsymbol{\theta}},\boldsymbol{\theta})} = \frac{1}{(2\pi)^{Nt}} \int \mathrm{d}\hat{\boldsymbol{\theta}} \mathrm{d}\mathbf{z} P(\mathbf{z}) \exp\left(\sum_{iu} \mathrm{i}\hat{\theta}_{i,u} (\theta_{i,u} - \beta H_{i,u} - \beta J_0 m_{u-1}) - \beta J_\sigma \sum_{i,uv} q_{u-1,v-1} \mathrm{i}\hat{\theta}_{i,u} z_{i,v} \right) \tag{319}$$

$$= \int \mathrm{d}\mathbf{z} P(\mathbf{z}) \prod_{iu} \delta\left(\theta_{i,u} - \hat{H}_{i,u}(\mathbf{z}) \right), \tag{320}$$

where $\hat{H}_{i,u}(\mathbf{z})$ is

$$\hat{H}_{i,u}(\mathbf{z}) = \beta H_{i,u} + \beta J_0 m_{u-1} + \beta J_\sigma \sum_v z_{i,v} q_{u-1,v-1}. \tag{321}$$

Here the distribution $P(\mathbf{z})$ is a multivariate Gaussian $\mathcal{N}(0, \boldsymbol{\Sigma})$, with $(\boldsymbol{\Sigma}^{-1})_{uv} = q_{u-1,v-1} = \frac{1}{N}\sum_i [\langle s_{i,u-1} s_{i,v-1} \rangle]$.

To simplify calculations, we can perform a change of variables

$$\tilde{z}_{i,u} = \sum_v z_{i,v} q_{u-1,v-1}. \tag{322}$$

Here, we can find that the covariance matrix of $\tilde{\boldsymbol{z}}_i$ is the inverse of the covariance matrix of $\boldsymbol{z}_i$. We can show this which we can describe using the characteristic function of the multivariate Gaussian $P(\mathbf{z})$

$$\phi_{\mathbf{z}_i}(\boldsymbol{\tau}) = \left\langle \exp\left(\mathrm{i} \sum_u \tau_u z_{i,u} \right) \right\rangle_{\mathbf{z}_i} = \exp\left(-\frac{1}{2} \sum_{uv} \tau_u \tau_v \Sigma_{uv} \right), \tag{323}$$

and computing then the characteristic function of $P(\tilde{\boldsymbol{z}}_i)$

$$\phi_{\tilde{\boldsymbol{z}}_i}(\boldsymbol{\tau}) = \left\langle \exp\left(\mathrm{i} \sum_u \tau_u \tilde{z}_{i,u} \right) \right\rangle_{\tilde{\boldsymbol{z}}_i} = \left\langle \exp\left(\mathrm{i} \sum_u \tau_u \sum_v z_{i,v} q_{u-1,v-1} \right) \right\rangle_{\mathbf{z}_i} \tag{324}$$

$$= \exp\left(-\frac{1}{2} \sum_{uu'} \tau_u \tau_{u'} \sum_{vv'} q_{u-1,v-1} \Sigma_{vv'} q_{u'-1,v'-1} \right) \tag{325}$$

$$= \exp\left(-\frac{1}{2} \sum_{uv} \tau_u \tau_v q_{u-1,v-1} \right), \tag{326}$$

where in the last step we took into account that $\boldsymbol{\tau}^T \mathbf{q}^T \boldsymbol{\Sigma} \mathbf{q} \boldsymbol{\tau} = \boldsymbol{\tau}^T \mathbf{q} \boldsymbol{\tau}$ as $\boldsymbol{\Sigma}^{-1} = \mathbf{q}$. This result indicates that $P(\tilde{\boldsymbol{z}}_i)$ is a multivariate Gaussian $\mathcal{N}(0, \mathbf{q})$.

After solving the saddle-point equation we have

$$\Phi(\mathbf{s},\boldsymbol{\theta}) = \sum_{iu} s_{i,u}\left(g_{i,u}+\theta_{i,u}\right) - \sum_{iu}\log 2\cosh\left(\theta_{i,u}\right), \tag{327}$$

which leads us to

$$[Z_t(\mathbf{g})] = \int \mathrm{d}\boldsymbol{\theta} \exp\left(\log \sum_{\mathbf{s}_{1:t}} \mathrm{e}^{\Phi(\mathbf{s},\boldsymbol{\theta})}\right) \int \mathrm{d}\tilde{\mathbf{z}} P(\tilde{\mathbf{z}}) \prod_{iu} \delta\left(\theta_{i,u} - \hat{H}_{i,u}(\tilde{\mathbf{z}})\right) \tag{328}$$

$$= \int \mathrm{d}\tilde{\mathbf{z}} P(\tilde{\mathbf{z}}) \exp\left(\log\left(\sum_{\mathbf{s}_{1:t}} \exp\left(\sum_{iu} s_{i,u}\left(g_{i,u}+\hat{H}_{i,u}(\tilde{\mathbf{z}})\right)\right) - \sum_{iu}\log 2\cosh\left(\hat{H}_{i,u}(\tilde{\mathbf{z}})\right)\right)\right) \tag{329}$$

$$= \int \mathrm{d}\mathbf{z} P(\tilde{\mathbf{z}}) \prod_{iu} \frac{\cosh\left(g_{i,u}+\hat{H}_{i,u}(\tilde{\mathbf{z}})\right)}{\cosh\left(\hat{H}_{i,u}(\tilde{\mathbf{z}})\right)}. \tag{330}$$

And, as the diagonal of the covariance matrix of $\tilde{\mathbf{z}}$ is equal to 1, we can derive

$$m_{i,u} = \lim_{\mathbf{g}\to\mathbf{0}} \frac{\partial Z_t(\mathbf{g})}{\partial g_{i,u}} = \int \mathrm{D}z \tanh(\hat{H}_{i,u}(z)), \tag{331}$$

$$R_{ii,uv} = \lim_{\mathbf{g}\to\mathbf{0}} \frac{\partial^2 Z_t(\mathbf{g})}{\partial g_{i,u}\partial g_{j,v}} = \int \mathrm{D}xy(q_{u-1,v-1}) \tanh(\hat{H}_{i,u}(x)) \tanh(\hat{H}_{i,v}(y)), \tag{332}$$

where

$$\mathrm{D}z = \frac{1}{\sqrt{2\pi}} \mathrm{e}^{-\frac{1}{2}z^2}, \tag{333}$$

$$\mathrm{D}xy(q) = \frac{1}{2\pi\sqrt{1-q^2}} \mathrm{e}^{-\frac{x^2+y^2-2qxy}{2(1-q^2)}}, \tag{334}$$

$$\hat{H}_{i,u}(z) = \beta H_{i,u} + \beta J_0 m_{u-1} + \beta J_\sigma z. \tag{335}$$

The resulting equations are similar to the symmetric SK model, but the replica-coupling parameter vanishes for the computation of m_u.

$$m_u = \frac{1}{N}\sum_i \int \mathrm{D}z \tanh\left(H_{i,u} + \beta J_0 m_{u-1} + \beta J_\sigma\right), \tag{336}$$

$$q_{uv} = \frac{1}{N}\sum_i \int \mathrm{D}xy(q_{u-1,v-1}) \tanh\left(H_{i,u} + \beta J_0 m_{u-1} + \beta J_\sigma x\right) \tanh\left(H_{i,v} + \beta J_0 m_{v-1} + \beta J_\sigma y\right). \tag{337}$$

This is consistent with findings of the asymmetric SK model lacking a spin-glass phase as m_u is independent of q_{uv}. Note also that all $q_{u,v}$ only depends on the previous $q_{u-1,v-1}$, this in the $t\to\infty$ limit the value of $q_{t,t-d}$ tends to the same value for any finite d.

Ferromagnetic critical phase transition in the infinite kinetic Ising model with Gaussian couplings and uniform weights

We define an Ising network of infinite size, with randomly defined bias $H_{i,u} = H_i$, where H_i has a distribution $\mathcal{U}(-H_0, H_0)$ and couplings J_{ij} with a Gaussian distribution $\mathcal{N}(\frac{1}{N}, \frac{J_\sigma^2}{N})$. We choose $H_0 = 0.5$, $J_\sigma = 0.1$.

As we have found that the asymmetric SK model with arbitrary fields follows a mean-field solution, calculating the effects of disorder in the fields becomes much easier, as we can approximate the update equations of the order parameters in the thermodynamic limit $N\to\infty$ as:

$$m_t = \sum_i m_{i,t} = \frac{1}{2H_0}\int_{-H_0}^{H_0} \mathrm{d}h \int \mathrm{D}z \tanh\left(\beta\left(h + J_0 m_{t-1} + J_\sigma z\right)\right) \tag{338}$$

$$= \frac{1}{2\beta H_0}\int \mathrm{D}z \log\left(\frac{\cosh\left(\beta\left(H_0 + J_0 m_{t-1} + J_\sigma z\right)\right)}{\cosh\left(\beta\left(-H_0 + J_0 m_{t-1} + J_\sigma z\right)\right)}\right). \tag{339}$$

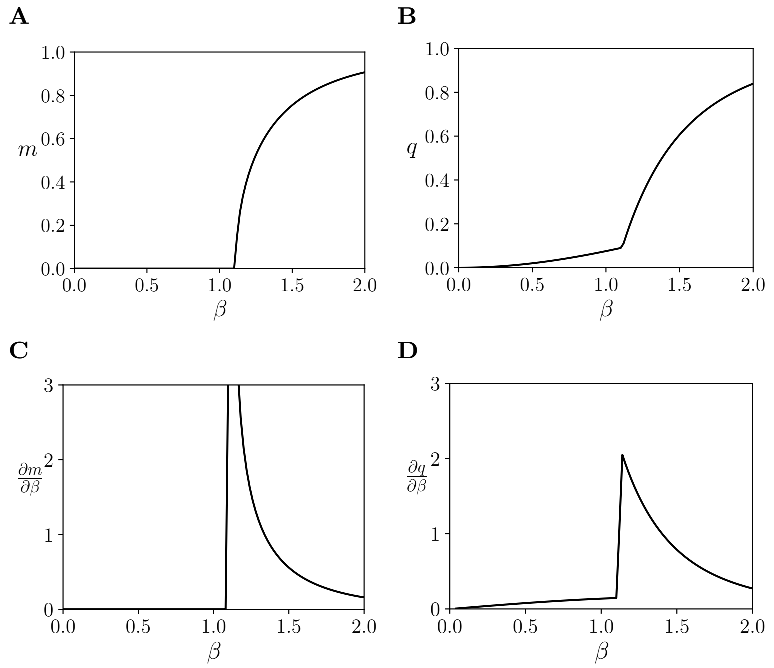

Supplementary Fig. 1. **Analytical results of the behaviour of the infinite kinetic Ising model**. A critical point is found for approximately $\beta_c = 1.1108$ with parameters $J_0 = 1$, $J_\sigma = 0.1$ and $H_0 = 0.5$.

Similarly, the delayed self-correlation parameter is given as

$$q_{t,t'} = \sum_i R_{ii,t,t'} = \frac{1}{2H_0}\int_{-H_0}^{H_0} \mathrm{d}h \int \mathrm{D}xy(q_{t-1,t'-1}) \tanh(h + J_0 m_{t-1} + J_\sigma x) \tanh(\beta(h + J_0 m_{t'-1} + J_\sigma y)) \tag{340}$$

$$= \frac{1}{2\beta H_0}\int \mathrm{D}xy(q_{u-1,v-1}) \left(\frac{1}{2} \left(\log \left(\frac{1+\tanh(H_0 + H_{t,x})}{1-\tanh(\beta(H_0+H_{t,x}))} \frac{1-\tanh(\beta(-H_0+H_{t,x}))}{1+\tanh(\beta(-H_0+H_{t,x}))} \right) \right) \right. \tag{341}$$

$$\left. - \frac{1}{\tanh\beta((J_0(H_{t',y} - H_{t,x}))} \log\left(\frac{1+\tanh\beta((H_{t',y}-H_{t,x}))\tanh(\beta(H_0+H_{t,x}))}{1+\tanh(\beta(H_{t',y}-H_{t,x}))\tanh(\beta(-H_0+H_{t,x}))} \right) \right). \tag{342}$$

with $H_{t,x} = J_0 m_{t-1} + J_\sigma x$ and $H_{t',y} = J_0 m_{t'-1} + J_\sigma y$.

By recursively updating Supplementary Eqs. 339 and 341-342, we can obtain the order parameters of the system. In Supplementary Fig. 1, we show the order parameters for $J_0 = 1$, $J_\sigma = 0.1$ and $H_0 = 0.5$, which resulted in a ferromagnetic transition around $\beta_c \approx 1.1108$.

Similarly, assuming a nonequilibrium steady state (NESS) in which $m_t = m_{t-1} = m$ we can obtain the critical point of the system by computing the non-zero solutions of the first order Taylor expansion around $m = 0$ of the right hand part of Supplementary Eq. 339,

$$m \approx \frac{1}{2\beta H_0}\int \mathrm{D}z \log\left(\frac{\cosh(\beta(H_0 + J_\sigma z))}{\cosh(\beta(-H_0+J_\sigma z))}\right) \tag{343}$$

$$+ \frac{1}{2\beta H_0}\int \mathrm{D}z\,(\tanh(\beta(H_0+J_\sigma z)) - \tanh(\beta(-H_0+J_\sigma z)))\,\beta J_0 m \tag{344}$$

$$= \frac{1}{H_0}\int \mathrm{D}z \tanh(\beta(H_0+J_\sigma z))\,J_0 m, \tag{345}$$

that yields the same solution for β_c solving the equation

$$\frac{1}{H_0}\int \mathrm{D}z \tanh(\beta_c(H_0+J_\sigma z))\,J_0 = 1. \tag{346}$$

We can characterize some critical exponents of the system using the reduced temperature $\tau = -\frac{\beta-\beta_c}{\beta_c}$ and computing the value of m around $\beta = \beta_c$ with the third order Taylor expansion

$$m \approx \frac{1}{H_0} \int \mathrm{D}z \tanh\left(\beta\left(H_0 + J_\sigma z\right)\right) J_0 m \tag{347}$$

$$- \frac{1}{3\beta H_0} \int \mathrm{D}z \tanh\left(\beta\left(H_0 + J_\sigma z\right)\right)\left(1 - \tanh^2\left(\beta\left(H_0 + J_\sigma z\right)\right)\right)\left(\beta J_0 m\right)^3 \tag{348}$$

$$= (1 - K'\tau)m - K''m^3, \tag{349}$$

$$m \propto (-\tau)^{-\frac{1}{2}}. \tag{350}$$

This yields a critical exponent $\beta' = \frac{1}{2}$ (note that this is different from the inverse temperature β), consistent with the mean-field universality class.

Similarly, we can compute the susceptibility to a uniform magnetic field B added on top of H_i, having that

$$\frac{\partial m}{\partial B} = \frac{1}{2\beta H_0} \int \mathrm{D}z \left(\tanh\left(\beta\left(H_0 + J_0 m + J_\sigma z\right)\right) - \tanh\left(\beta\left(-H_0 + J_0 m + J_\sigma z\right)\right)\right)\left(\beta + \beta J_0 \frac{\partial m}{\partial B}\right). \tag{351}$$

The susceptibility evaluated at $m = 0$ yields, for the limit $\tau \to 0$,

$$\frac{\partial m}{\partial B} = (1 - K'\tau)\left(\frac{1}{J_0} + \frac{\partial m}{\partial B}\right), \tag{352}$$

$$\frac{\partial m}{\partial B} \propto \frac{1 - K'\tau}{\tau} \approx (-\tau)^{-1}. \tag{353}$$

The result retrieves the $\gamma = 1$ exponent consistent with the mean-field universality class.

Finite-size fluctuations

Finally, to evaluate the behaviour around β_c in the finite network used in the article, we numerically simulated the behaviour of a network of size $N = 512$, with parameters $H_0 = 0.5$, $J_0 = 1$, $J_\sigma = 0.1$ as in the numerical results from the main text. In Supplementary Fig. 2 we show the values of $\mathbf{m}_t$. $\mathbf{C}_t$ and $\mathbf{D}_t$, showing that correlations peak around the value of β_c computed theoretically.

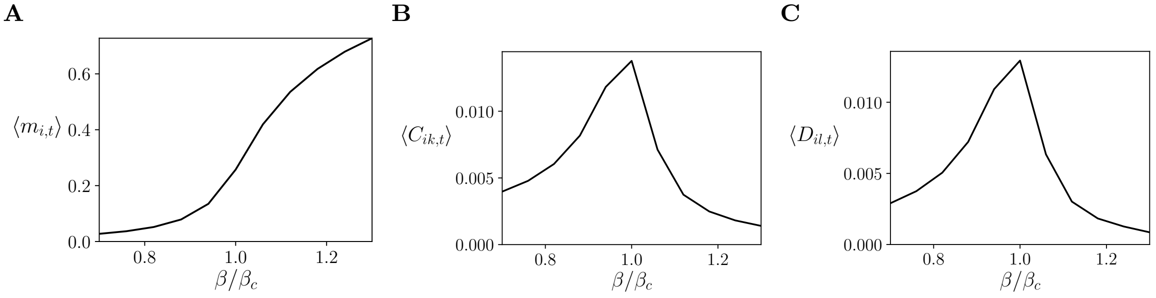

Supplementary Fig. 2. **Finite-size simulation of the asymmetric Ising model**. Experimental results of the behaviour around the critical point ($\beta_c = 1.1108$) of an asymmetric Ising model with $N = 512$ and parameters $H_0 = 0.5$, $J_0 = 1$ and $J_\sigma = 0.1$.

Supplementary Note 7: Complexity of the models and their computational costs

Here we clarify the structure of each model, and quantitatively compare the relation between model complexity and their computational cost. We start by noting that the reference model in Plefka[$t-1, t$] is a submodel of the reference models in the other three approximation models used in Plefka[t], Plefka[$t-1$], and Plefka2[t]. The latter approximation models (Plefka[t], Plefka[$t-1$], Plefka2[t]) are not mutually inclusive, rather each model manifests a distinct assumption.

To clarify the relation between the performance of each approximation and its model complexity, we provide the number of free and fixed parameters of each model. We then examine the relation between accuracy of the models in forward/inverse Ising problems and their computational time required to solve these problems.

Number of free and fixed parameters in the models

To compare the structure of each approximated model, let us first revisit the definition of each model, and introduce the number of their free or fixed parameters. Each approximation of P (a marginal distribution of spins covering some time steps t of the model) defines a reference manifold using Q to perform a Plefka expansion. Q is defined at each time point, and obtained by fixing some parameters (couplings) of P at zero. The rest of the parameters are either free parameters that are fitted as an m-projection from P, or preserved as the original values of P. In the Supplementary Table I, we listed the number of free and fixed (either to zero or to their original value) parameters of reference models. For example, in Plefka[t], we preserve one field parameter (H_i) and N couplings (J_{ij}) for each i-th spin at time $t-1$ with their original values, while fixing N couplings of the i-th spin at time t at zero. A field parameter of the i-th neuron at time t ($\Theta_{i,t}$) is a free parameter obtained as an m-projection from P (i.e., it is fitted so that Q and P have the same expectation $\mathbf{m}$). We need to perform this projection for all neurons (i.e., N times).

	Number of times the approximation is computed	Free parameters to be fitted (at each approximation)	Parameters fixed at zero (at each approximation)	Parameters fixed at their original value (for each approximation)
Plefka[$t-1, t$]	$2N$ (N neurons at time t and N neurons at time $t-1$)	1 field	N couplings	0
Plefka[t]	N (N neurons at time t)	1 field	N couplings	1 field + N couplings (for N neurons at $t-1$)
Plefka[$t-1$]	N (N neurons at time $t-1$)	1 field	N couplings	1 field + N couplings (for N neurons at t)
Plefka2[t]	$2N^2$ (once for every pair of neurons at time t once for every pair at $t, t-1$)	2 fields (time t and $t-1$) and 1 coupling	$N-1$ couplings at t, N couplings at $t-1$	1 field and N couplings (for $N-1$ neurons except for l at $t-1$)

Supplementary Table I. **Comparison of methods**. The number of free and fixed parameters of each approximation model.

Accuracy versus computational costs

The structure of the reference model Q influences both the accuracy of the approximation methods and their computational complexity. For example, Plefka[t] is computationally more demanding than Plefka[$t-1, t$] since the former requires to compute multiplications over the correlation matrices $\mathbf{C}_{t-1}$, while the latter only makes use of the means $\mathbf{m}_{t-1}$. Plefka[$t-1$] involves performing a Gaussian integral, which becomes computationally demanding, especially for the equal-time correlations, where one needs to perform a 2-dimensional integral. Plefka2[t] is computationally more expensive than Plefka[t] because, while the latter needs to be computed N times (once per spin), the former needs to be computed $2N^2$ times (once per each pair of delayed and same-time spins).

In Supplementary Fig. 3, we show the trade-off between computational time and accuracy for the different methods. These results are obtained by simulating the model for $T = 128$ steps at $\beta = \beta_c$ (in principle the most challenging point of our model). We computed the average squared error of its statistical moments and cumulants $\epsilon_{\mathbf{m}} = \langle\langle(m^o_{i,t} - m^p_{i,t})^2\rangle_i\rangle_t$, $\epsilon_{\mathbf{C}} = \langle\langle(C^o_{ik,t} - C^p_{ik,t})^2\rangle_{ik}\rangle_t$ and $\epsilon_{\mathbf{D}} = \langle\langle(D^o_{ik,t} - D^p_{ik,t})^2\rangle_{il}\rangle_t$.

As expected, Plefka$[t-1,t]$ is the fastest method, although it yields the most inaccurate approximation. Plefka$[t]$ significantly improves accuracy with a slight increase in computational time. Plefka2$[t]$ increases further both computational cost and accuracy. Finally, Plefka$[t-1]$ is relatively slow compared to the other methods, offering a poor performance in the forward Ising problem. In sum, we recommend to use Plefka2$[t]$ if users pursue the accuracy, and Plefka$[t]$ if users pursue the speed in the forward Ising problem.

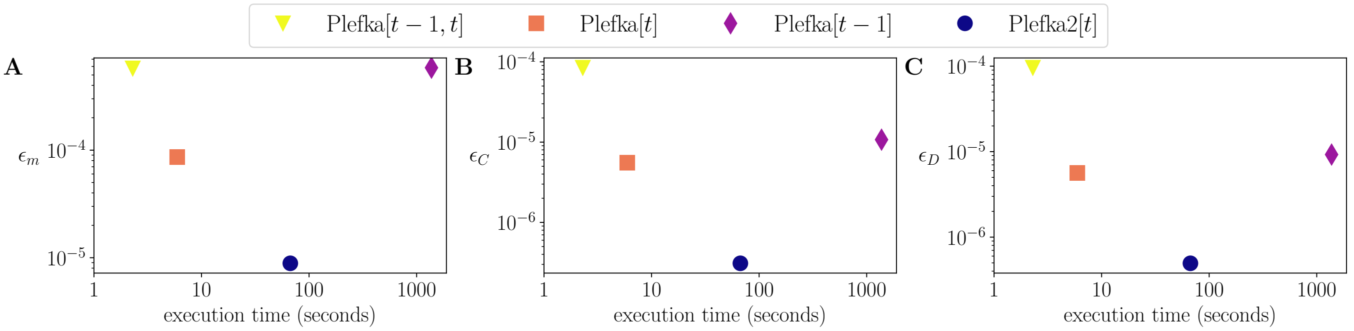

Supplementary Fig. 3. **Accuracy vs computational time in the forward Ising problem**. Average squared error in (A) the Ising model average magnetizations, (B) equal-time correlations and (C) delayed correlations at $t = 1, \ldots, 128$ versus computation time in minutes for different mean-field models for $\beta = \beta_c$. Results were obtained using a 2-core Intel(R) Core(TM) i7-5500U CPU @ 2.40GHz processor.

In Supplementary Fig. 4, we show the results for the inverse Ising problem at $\beta = \beta_c$. The (in)accuracy is evaluated by the average squared error between the inferred parameters and true parameters: $\epsilon_{\mathbf{H}} = \langle\langle(H^o_{i,t} - H^p_{i,t})^2\rangle_i\rangle_t$ and $\epsilon_{\mathbf{J}} = \langle\langle(J^o_{ik,t} - J^p_{ik,t})^2\rangle_{il}\rangle_t$. We found that the results are different from the forward Ising model. In the inference problem, Plefka$[t-1,t]$ shows a poor performance in accuracy as in the forward problem, but it was no longer the fastest method. Plefka$[t]$, Plefka$[t-1]$ and Plefka2$[t]$ offer similar performances.

There are several reasons why the results in the inverse Ising problem differ from the previous forward problem. First, the computational time in the inverse problem depends on how fast the learning dynamics reach convergence. These dynamics differ across the methods (e.g., due to the slowest speed of convergence, Plefka$[t-1,t]$ is slightly slower than Plefka$[t]$). In addition, we need to compute only $\mathbf{m}_t$ and $\mathbf{D}_t$, and do not need to compute $\mathbf{C}_t$ in the inverse problem. Since Plefka$[t-1]$ is very slow in computing $\mathbf{C}_t$, by freeing from its computational load, Plefka$[t-1]$ becomes much faster in the inverse problem.

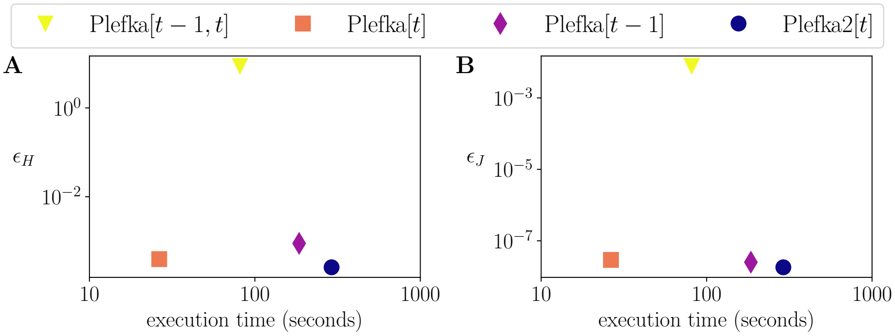

Supplementary Fig. 4. **Accuracy vs computational time in the inverse Ising problem**. Average squared error in the inverse Ising inferred (A) external fields and (B) couplings versus computation time in minutes for different mean-field models for $\beta = \beta_c$. Results were obtained using a 2-core Intel(R) Core(TM) i7-5500U CPU @ 2.40GHz processor.

[1] H. J. Kappen and J. J. Spanjers, Mean field theory for asymmetric neural networks, Physical Review E **61**, 5658 (2000).

[2] Y. Roudi and J. Hertz, Dynamical TAP equations for non-equilibrium Ising spin glasses, Journal of Statistical Mechanics: Theory and Experiment **2011**, P03031 (2011).

[3] Y. Roudi and J. Hertz, Mean Field Theory for Nonequilibrium Network Reconstruction, Physical Review Letters **106**, 048702 (2011).

[4] M. Mézard and J. Sakellariou, Exact mean-field inference in asymmetric kinetic Ising systems, Journal of Statistical Mechanics: Theory and Experiment **2011**, L07001 (2011).

[5] H. Nishimori, *Statistical physics of spin glasses and information processing: an introduction* (Clarendon Press, 2001).

[6] R. Brunetti, G. Parisi, and F. Ritort, Asymmetric Little spin-glass model, Physical Review B **46**, 5339 (1992).

[7] V. Lecomte, C. Appert-Rolland, and F. van Wijland, Thermodynamic Formalism for Systems with Markov Dynamics, Journal of Statistical Physics **127**, 51 (2007), zSCC: 0000223.
